# Broad phylogenetic and functional diversity among mixotrophic consumers of *Prochlorococcus*

**DOI:** 10.1038/s41396-022-01204-z

**Published:** 2022-02-10

**Authors:** Qian Li, Kyle F. Edwards, Christopher R. Schvarcz, Grieg F. Steward

**Affiliations:** 1 School of Oceanography, Shanghai Jiao Tong University, Shanghai, China; 2 Department of Oceanography, School of Ocean and Earth Science and Technology (SOEST), University of Hawaiʻi at Mānoa, Honolulu, HI USA; 3 Daniel K. Inouye Center for Microbial Oceanography: Research and Education, School of Ocean and Earth Science and Technology (SOEST), University of Hawaiʻi at Mānoa, Honolulu, HI USA

**Keywords:** Microbial biooceanography, Microbial ecology, Biodiversity, Molecular ecology

## Abstract

Small eukaryotic phytoplankton are major contributors to global primary production and marine biogeochemical cycles. Many taxa are thought to be mixotrophic, but quantitative studies of phagotrophy exist for very few. In addition, little is known about consumers of *Prochlorococcus*, the abundant cyanobacterium at the base of oligotrophic ocean food webs. Here we describe thirty-nine new phytoplankton isolates from the North Pacific Subtropical Gyre (Station ALOHA), all flagellates ~2–5 µm diameter, and we quantify their ability to graze *Prochlorococcus*. The mixotrophs are from diverse classes (dictyochophytes, haptophytes, chrysophytes, bolidophytes, a dinoflagellate, and a chlorarachniophyte), many from previously uncultured clades. Grazing ability varied substantially, with specific clearance rate (volume cleared per body volume) varying over ten-fold across isolates and six-fold across genera. Slower grazers tended to create more biovolume per prey biovolume consumed. Using qPCR we found that the haptophyte *Chrysochromulina* was most abundant among the isolated mixotrophs at Station ALOHA, with 76–250 cells mL^−1^ across depths in the upper euphotic zone (5–100 m). Our results show that within a single ecosystem the phototrophs that ingest bacteria come from many branches of the eukaryotic tree, and are functionally diverse, indicating a broad range of strategies along the spectrum from phototrophy to phagotrophy.

## Introduction

Small eukaryotic phytoplankton in the ‘pico’ (<3 µm) and ‘nano’ (2–20 µm) size classes are often dominant contributors to phytoplankton biomass and primary production in open oceans [1–3] and are major drivers of nutrient cycling [4, 5]. At the same time, many flagellate and ciliate taxa are known to be mixotrophic, capable of obtaining nutrition through combined photosynthesis (autotrophy) and phagocytosis (heterotrophy) [6, 7]. Mixotrophs have been observed in sunlit habitats throughout the ocean [8, 9] and are estimated to contribute about two thirds of total bacterivory in open-ocean Atlantic ecosystems [10]. Phototrophic and heterotrophic bacteria are themselves major contributors to pelagic production and biomass [11–13], and therefore protists that can both photosynthesize and prey on prokaryotes may play a key role in regulating oceanic productivity, element cycling, and food web dynamics. To better understand these processes, more quantitative data is needed on grazing kinetics and growth efficiencies of the predominant grazers of prokaryotes. It may be particularly consequential if the main grazers are mixotrophs, because models predict that mixotrophic consumers increase primary production and carbon export, and decrease nutrient remineralization, relative to heterotrophic consumers [14, 15].

Although the aggregate importance of pigmented flagellates for bacterial grazing has been documented [16, 17], much less is known about which taxa are the major grazers, and how the broad phylogenetic diversity among these organisms translates into diverse ecological roles [8, 18]. Progress in this area has been impeded in part by a paucity of representative cultured mixotrophs relative to the complexity found in natural communities [18, 19], which often contain haptophytes, chrysophytes, dictyochophytes, chlorophytes, bolidophytes, cryptophytes, and dinoflagellates [20].

The North Pacific Subtropical Gyre (NPSG) is a chronically oligotrophic environment, where prokaryotic and eukaryotic phototrophs are dominated by *Prochlorococcus* [21] and small flagellates [22], respectively. Some of the likely grazers of *Prochlorococcus* in this habitat were identified by stable isotope probing by addition of ^13^C- and ^15^N-labeled *Prochlorococcus* MED4 cells to natural communities [23]. The 18S rRNA of various haptophytes, dictyochophytes, bolidophytes, and dinophytes became significantly labeled, but most of the specific phylotypes identified in that study have not been isolated, and controlled lab studies of their grazing capabilities have been lacking. There has thus been no confirmation that most of these organisms directly ingest *Prochlorococcus* and no quantitative assessment of how ingestion rates and functional responses vary among them.

One exception is a recent study of the grazing ecophysiology of a phagotrophic mixotroph *Florenciella* (strain UHM3021; class Dictyochophyceae) isolated from the NPSG [24]. Given sufficient light, rapid growth of *Florenciella* was sustained by feeding on bacteria (*Prochlorococcus, Synechococcus*, or a heterotrophic bacterium) as the primary nutrient source, suggesting mixotrophy is an effective strategy for nutrient acquisition. The rate at which prey were ingested by this *Florenciella* strain was relatively low compared to heterotrophic flagellates of similar size, and prey ingestion was suppressed by high concentrations of dissolved nutrients. This was the first detailed characterization of *Prochlorococcus* consumption by a mixotrophic flagellate, and it remains unclear whether other small, open-ocean mixotrophs possess similar physiology. For example, *Florenciella* may be a relatively autotrophic mixotroph that relies on prey primarily for limiting nutrients [25]; other taxa may be more voracious grazers that rely on prey as a major energy source [26, 27].

To broaden our understanding of how mixotrophs feed on one of the most important primary producers in the ocean, we isolated diverse mixotrophs (dictyochophytes, haptophytes, chrysophytes, bolidophytes, a dinoflagellate, and a chlorarachniophyte) from Station ALOHA, an open-ocean site in the NPSG, 100 km north of the island of O’ahu. By characterizing their grazing capabilities using *Prochlorococcus* as prey, and by quantifying their in situ abundances, we reveal functional diversity among the mixotrophs in this ecosystem and their contributions to *Prochlorococcus* mortality in situ.

## Methodology

### Isolation and cultivation

In total, 39 mixotrophic flagellates were investigated (Table 1). Thirty-three isolates were enriched and isolated from euphotic zone samples at Station ALOHA (22° 45′ N, 158° 00′ W) in February and May 2019. To select for mixotrophic grazers of *Prochlorococcus*, whole seawater was amended with K medium [28] (1/20 final concentration), and live *Procholorococcus* (MIT9301) was added as prey (~5 × 10^6^ cells mL^−1^ final concentration). Enriched seawater samples were incubated under ~70 µmol photons m^−2^ s^−1^ irradiance on a 12 h:12 h light:dark cycle and monitored by microscopy daily up to five days. Each day, samples were serially diluted to extinction (9 dilution steps, 12 replicates per dilution) in 96-well plates in nutrient-reduced K medium (1/20 concentration) with a constant background of *Prochlorococcus* cells. Wells at the highest dilution showing growth of putative grazers were subjected to 3–6 further rounds of dilution to extinction. Four additional mixotrophs were isolated in full K medium using water from earlier cruises, and two (dictyochophyte strains UHM3021 described in [24] and UHM3050) were enriched in K minus nitrogen medium (K-N) without *Prochlorococcus* enrichment. All isolates were rendered unialgal, but not axenic, and maintained at 24 °C in K-N medium (~0.2 µM N) amended with *Prochlorococcus* prey, under the same light conditions as above. Dense *Prochlorococcus* cells grown in Pro99 medium [29], were harvested and concentrated through gentle centrifugation at 2000 RCF for 5 minutes and resuspended in fresh K-N medium to minimize nutrient carryover. To ensure their long-term accessibility, the isolates used in this study are being transferred to the National Center for Marine Algae and Microbiota at the Bigelow Laboratory for Ocean Science, East Boothbay, ME, USA (ncma.bigelow.org).

**Table 1:** Mixotrophic flagellates investigated in this study.

Phylum	Class	Genus/Clade^a^ (abbreviation)	Isolates	Source depths (m)	Collection dates (yyyy/mm)	ESD^b^ (µm)	Number tested for grazing rate^c^	Number tested for functional response^d^
Ochrophyta	Dictyochophyceae	*Florenciella*	19	25, 75, 100	2011/03, 2014/09, 2019/02, 2019/05	3.0–4.3	12	1
		*Rhizochromulina*	4	25, 75	2019/05	3.0–3.2	4	1
		Dictyochophyceae-X (DictyX)	6	25, 100	2015/02, 2019/02, 2019/05	4.0–5.3	5	1
	Bolidophyceae	*Triparma*	2	25, 100	2019/05	2.5–2.7	2	0
	Chrysophyceae	Chrysophyceae-H (ChrysoH)	3	25	2019/05	2.2–2.4	3	1
Haptophyta	Prymnesiophyceae	*Chrysochromulina*	2	5, 45	2012/01, 2015/08	3.8–4.3	2	1
	unclassified	Haptophyta-2 (Hap2)	1	25	2019/02	4.5	1	1
Cercozoa	Chlorarachniophyceae	Chlorarachniophyceae-X (ChloraX)	1	n/a	2011/03	3.8	1	0
Myzozoa	Dinophyceae	*Pelagodinium*	1	25	2019/05	4.0	1	0

^a^DictyX, Hap2, ChrysoH, and ChloraX are abbreviations for the undescribed or environmental clades of Dictyochophyceae-X, Haptophyta clade HAP-2, Chrysophyceae clade H, and Chlorarachniophyceae-X, respectively, which cluster at approximately the genus level.

^b^*ESD* equivalent spherical diameter.

^c^Number of individual isolates of each type for which grazing rates were calculated at one or more prey concentration. Eight additional isolates (seven *Florenciella*, one DictyX) were observed to consume *Prochlorococcus*, but rates were not calculated, because of low sampling frequency.

^d^Number of isolates of each type for which grazing rates were measured at multiple prey concentrations to determine the grazing functional response and estimate grazing parameters *C*_max_ and *I*_max_.

### 18S rRNA gene sequencing and phylogenetic analysis

Cells were harvested by centrifuging 25–50 mL dense cultures at 3000 RCF for 10 min at 4 °C. Genomic DNA was extracted from the pellets using the ZymoBIOMICS DNA Kit (Zymo Research, Irvine, CA, USA). A near-full-length section of the eukaryotic small-subunit ribosomal RNA (18S rRNA) gene was amplified by PCR with the Roche Expand High Fidelity PCR System (Sigma-Aldrich, St. Louis, MO, USA) using either forward primer 5′-ACCTGGTTGATCCTGCCAG-3′ and reverse primer 5′-TGATCCTTCYGCAGGTTCAC-3′ [30], or Euk63F 5′-ACGCTTGTCTCAAAGATTA-3 and Euk1818R 5′-ACGGAAACCTTGTTACGA-3′ [31]. Amplicons were purified using spin columns (DNA Clean & Concentrator-25; Zymo Research, Irvine, CA, USA) and sequenced (Sanger) using the same PCR amplification primers and an additional reverse primer 1174R, 5′-CCCGTGTTGAGTCAAA-3′ [32], when necessary to connect two ends. For phylogenetic analyses, similar sequences were retrieved from the PR^2^ database [33] based on BLAST similarity, and two environmental homologs (GenBank Acc. FJ537342 and FJ537336) were retrieved from NCBI GenBank for the undescribed haptophyte taxon, which was not affiliated with any reference sequence from the PR^2^ database. Sequence alignments including 39 isolates, 29 reference and 2 outgroup taxa were created with MAFFT v7.450 using the G-INS-i algorithm [34] in Geneious R11.1.5 (http://www.geneious.com) [35]. Terminal sites that lacked data for any of the sequences were trimmed and any sites with greater than 25% gaps were removed from the alignment, which generated a total sequence length of 1617 bases. Phylogenetic analysis was performed using MrBayes v3.2.6 in Geneious R11.1.5 [36] with two runs of four chains for 1,000,000 generations, subsampling every 200 generations with burn-in length 100,000, under the GTR substitution model. The Bayesian majority consensus tree was further edited within iTOL v5 [37]. All 18 S rRNA gene sequences were deposited in GenBank with accession numbers MZ611704–MZ611740; MN615710–MN615711.

### Microscopic observation

The average diameter of flagellates in the exponential growth phase (*n* = 20 cells per strain) was measured by transmitted light microscopy using image analysis software (NIS-Elements AR, Nikon, Minato City, Tokyo, Japan) calibrated with a stage micrometer. Equivalent spherical diameter (ESD) and biovolumes were calculated assuming spherical cells. Chloroplasts were visualized by autofluorescence under epifluorescence microscopy. An average ESD of 0.64 µm was used for *Prochlorococcus* prey [24].

Visual evidence of phagocytosis was obtained by adding fluorescent beads (0.5 μm YG Fluoresbrite Microspheres; Polysciences) to each culture. Samples post incubation (~2 h) were fixed with an equal volume of 4% ice-cold glutaraldehyde, and subsamples (20 µL) were mounted on a glass slide under a coverslip. Paired images captured using epifluorescence and transmitted light microscopy (Olympus BX51 with Leica DFC 7000 T color digital camera) were overlain to identify cells with ingested beads.

### Grazing experiments

Long-term grazing experiments were conducted for all 39 grazers, and 31 were used to quantify grazing rates based on rates of disappearance of *Prochlorococcus* cells, which persist but do not readily grow in K-N medium [24]. Rates were not calculated for eight isolates (seven *Florenciella* and one DictyX) because they were sampled at a lower frequency. Fifteen isolates representing all genera (or approximately genus-level clades) were examined in more detail by replicating grazing experiments two times (marked in bold in Supplementary Table S1), while the remaining sixteen isolates were tested once to survey within- and across- genus variation. Prior to the experiments, all grazer cultures were maintained/acclimated in the experimental medium (K-N with prey). Experiments were initialized by inoculating late-exponential-phase grazers into fresh K-N medium at a final concentration of ~10^3^ flagellates mL^−1^, and adding live, unstained prey at a final concentration of 2–3 × 10^6^ *Prochlorococcus* mL^−1^. Grazers were incubated for 3–8 days in total, depending on how fast prey were ingested. To minimize carryover of dissolved nutrients and prey growth in the grazing experiments, *Prochlorococcus* were grown to stationary phase in Pro99 medium, then pelleted (2000 RCF for 3–5 min) and resuspended in fresh K-N medium prior to addition to control and experimental cultures. Control cultures of grazer without added prey and prey without grazers were included during each grazing experiment to confirm that grazer growth and prey removal were attributable to grazing. Cell concentrations of prey and grazers were measured every 12–24 h by flow cytometry of glutaraldehyde-fixed samples at final concentration of 0.5% (Attune NxT; Thermo Fisher Scientific, Waltham, MA, USA, and CytoFLEX; Beckman Coulter Life Sciences, Indianapolis, IN, USA). Populations were distinguished based on light scatter and pigment autofluorescence and occasionally confirmed with DNA fluorescence (stained post-sampling with DNA stain SYBR Green I). Ambient bacteria concentrations, monitored using DNA stains, were ≤10% of the added *Prochlorococcus* at the start and during most periods of all grazing experiments.

### Grazing rates and biovolume conversion efficiency

We calculated ingestion rates, *I* (prey grazer^−1^ h^−1^) for each grazer as:

1 $$I = \frac{{P_t - P_{t + 1}}}{{G_{{{{{\rm{avg}}}}}}(T_{t + 1} - T_t)}}$$

where $P_t$ and $P_{t + 1}$ are the prey abundance at sampling interval *t* and *t* + 1 (cells mL^−1^), *G*_avg_ is the arithmetic mean grazer abundance (cells mL^−1^) over the time interval, and $(T_{t + 1} - T_t)$ is the time (h) between two sampling intervals. Clearance rates (*C*, nL grazer^−1^ h^−1^) were calculated by dividing the ingestion rate by average prey concentration over the same interval, and specific clearance rate (body volume grazer^−1^ h^−1^) was calculated by dividing the clearance rate by cellular biovolume (µm^3^) of each grazer. Equation (1) uses a linear approximation of prey and grazer trajectories over the sampling interval, which was appropriate for our data where change could be relatively linear, concave-up, or concave-down. Other commonly used ingestion rate calculations assume exponential prey decline [38] and/or exponential grazer growth [39].

Clearance rates over time in each experiment were assessed visually to obtain a representative series of rates that minimized potential influence of modest prey growth/decline observed in control cultures (Supplementary Fig. S1), as well as potential slowing of ingestion as the grazer neared carrying capacity or depleted prey to a low concentration. For each experiment a contiguous set of relatively constant rates were used to calculate a mean clearance rate. This assessment sometimes excluded the first 12–24 h, but not when removal rates were particularly fast. Intervals when the grazer neared carrying capacity were also often excluded, if grazing rates slowed down. To assess whether clearance rates increased as prey were depleted we plotted clearance rate and ingestion rate as a function of prey concentration (Supplementary Figs. S2 and S3). In general, there was no relationship between clearance rate and prey concentration, and ingestion increased linearly with prey concentration. These patterns imply that the prey concentrations in this experiment did not saturate the ingestion rates of these grazers. Under non-saturating prey concentrations the average clearance rate over an experiment should be a good estimate of the maximum clearance rate (*C*_max_). Consistent with this interpretation, functional responses fit to these experiments yielded *C*_max_ estimates that were similar to the reported average clearance rates. Because these experiments were not designed with a sufficient range of prey density to esimate functional responses, we do not report the *C*_max_ estimates, but we note here that our reported average clearance rates may be useful as approximate *C*_max_ numbers in future work.

Six grazers representing three classes (three dictyochophytes, two haptophytes, and one chrysophyte) were further investigated to determine functional grazing responses using a wide range of initial prey densities (10^5^–10^7^ cells mL^−1^). Functional responses were modeled using the Holling type II curve, $I = \frac{{I_{{{{{\rm{max}}}}}}P}}{{P + \frac{{I_{{{{{\rm{max}}}}}}}}{{C_{{{{{\rm{max}}}}}}}}}}$, where *I* is the ingestion rate over a sampling interval (Eq. 1), *I*_max_ is the maximum ingestion rate, *C*_max_ is the maximum clearance rate and *P* is the arithmetic mean prey density between two sampling points. This curve was fit to ingestion rate data using maximum likelihood with R package bbmle [40].

For 31 isolates we calculated the amount of grazer biovolume created per prey biovolume consumed, using data from the same grazing experiments used to calculate grazing rates. It was calculated based on the following formula:

2 $$E = \frac{{(F_{{{{{\rm{f}}}}}} - F_{{{{{\rm{i}}}}}})}}{{(P_{{{{{\rm{i}}}}}} - P_{{{{{\rm{f}}}}}})}}\frac{{B_{{{{{\rm{F}}}}}}}}{{B_{{{{{\rm{p}}}}}}}}$$

where *F*_f_ and *F*_i_ are the final and initial flagellate concentrations, *P*_i_ and *P*_f_ are initial and final prey concentrations in each culture, and *B*_*F*_ and *B*_P_ are the cellular biovolume of prey and grazer. We refer to the quantity *E* as the *biovolume conversion efficiency*, and we use it as an indicator of physiological differences among diverse mixotrophs. Note that biovolume conversion efficiency can be greater than 1, if prey have greater nutrient:biovolume than the grazer.

### Quantitative PCR

Real-time, quantitative PCR (qPCR) was performed to quantify the 18S rRNA gene abundances of representative mixotroph groups discriminated at approximately the genus level, including *Florenciella*, *Rhizochromulina* and another undescribed clade within the class Dictyochophyceae; *Chrysochromulina* and another undescribed clade within the division Haptophyta; clade H in the class Chrysophyceae*;* and *Triparma eleuthera* and *Triparma mediterranea* in the class Bolidophyceae. Primers (Supplementary Table S2) were designed to target a short region (95–176 bases) of the 18S rRNA gene and meet basic criteria (≤2 °C difference in melting temperature between members of a pair, %G + C content between 45 and 65%, ≤1 degenerate position per primer, no predicted primer dimers). Sequences considered targets for a given primer set had ≤1 mismatch across both primers, which included all or most known members within the corresponding targeted clade. Members in the nearest non-targeted clade had ≥3 mismatches distributed across both primers. Efficiency and specificity of the synthesized primers (IDT Inc., Coralville, IA, USA) was tested by ensuring there was specific amplification (qPCR followed by melting curve analysis and gel electrophoresis) when using DNA from cultures within the targeted group and no amplification when using DNA from cultures close to, but outside of the targeted group (Supplementary Table S3). Empirical observations of amplification success using control cultures were used to infer whether species known only by environmental sequences were likely to amplify with a given primer set (Supplementary Fig. S4).

In situ gene abundances were quantified in water samples collected from Station ALOHA at 5, 25, 45, 75, 100, 125, 150, and 175 m, during HOT cruise numbers 259 (Jan), 262 (Apr), 264 (Jul), and 266 (Oct) of 2014. Seawater (ca. 2 L) was filtered through 0.02 μm pore-size, aluminum oxide filters (Whatman Anotop, Sigma-Aldrich, Saint Louis, MO, USA) and stored at −80 °C. Genomic DNA of both grazer cultures and environmental samples was extracted (MasterPure Complete DNA and RNA Purification Kit; Epicentre) as described elsewhere [41]. Four replicated PCR reactions (10 μL) were carried out for each sample except for *Triparma* (duplicates) and consisted of 5 μl of 2× PowerTrack SYBR Green Master Mix (Thermo Fisher Scientific, USA), 10 ng environmental DNA, 500 nM of each primer, and nuclease-free water. Reactions were run on an Eppendorf Mastercycler epgradient S realplex^2^ real-time PCR instrument. Each run contained fresh serial dilutions (1–6 log gene copies) of target-specific, 750-bp synthetic standards (gBlocks, IDT) prepared in triplicate. The cycling program included an initial denaturation step of 95 °C for 2 min, followed by 40 cycles of 95 °C for 5 s and 55 °C for 30 s. Specificity of amplification was checked with a melting curve run immediately after the PCR program and occasionally, by gel electrophoresis. Amplification efficiencies ranged from 95% to 106% for all the primers.

To convert gene copies to cell numbers, 18S rRNA gene copy number per cell^−1^ was determined for representative isolates in the seven targeted genera/clades. Known quantities of cultured cells (10^6^–10^7^ cells) from each isolate with 2–8 replicates were pelleted at 4000 RCF for 15 min at 4 °C. DNA was extracted from the pelleted cells (MasterPure Complete DNA and RNA Purification Kit, Lucigen), quantified by fluorometry (Qubit, Invitrogen) and the extract volume adjusted to achieve a DNA concentration of 10 ng µL^−1^. The expected number of eukaryotic cells µL^−1^ of extract was calculated as the difference between the total cells in the sample prior to centrifugation and in the supernatant afterward (as determined by flow cytometry) divided by the final extract volume. Copy number of the 18S rRNA genes µL^−1^ of extract was determined by qPCR with the appropriate group-specific primers. The resulting value of gene copies µL^−1^ was divided by the equivalent number of eukaryotic cells µL^−1^ in the extract (assuming 100% extraction efficiency) to derive minimum estimates of gene copies cell^−1^. An average value for representatives within each genus/clade (1–5 isolates) was used to calculate in situ cell concentrations for the genus. These derived in situ abundances were compared to flow cytometric counts of total photosynthetic picoeukaryotes at Station ALOHA obtained from the Hawai’i Ocean Time-series Data Organization and Graphical System (https://hahana.soest.hawaii.edu/hot/hot-dogs/).

### Global distribution revealed through *Tara* Oceans 18S rRNA metabarcodes

To estimate the relative abundance of the OTUs closely related to our diverse isolates on a broader geographic scale, we searched the 18S rRNA-V9 sequence data from the 0.8–5 µm fraction of surface water sampled at 40 stations by the *Tara* Oceans project (http://taraoceans.sb-roscoff.fr/EukDiv/). Reads for ‘*Tara* lineages’ with highest similarity (*E*-value < 10^−15^) to each of our targeted clades (Supplementary Table S1) were expressed as a fraction of total reads excluding dinoflagellates but included all other *Tara* Oceans phytoplankton ‘taxogroups’: Bacillariophyta, Bolidophyceae, Chlorarachnea, Chlorophyceae, Chrysophyceae/Synurophyceae, Cryptophyta, Dictyochophyceae, Euglenida, Glaucocystophyta, Haptophyta, Mamiellophyceae, Other Archaeplastida, Other Chlorophyta, Pelagophyceae, Phaeophyceae, Pinguiophyceae, Prasino-Clade-7, Pyramimonadales, Raphidophyceae, Rhodophyta and Trebouxiophyceae. Dinoflagellates were excluded because of the difficulty in assigning phototrophic vs. heterotrophic status to all taxa, and because nearly all dinoflagellate reads were from a single, poorly annotated OTU that was also highly abundant in larger size fractions.

## Results

### Phylogenetic diversity

**Fig. 1:**
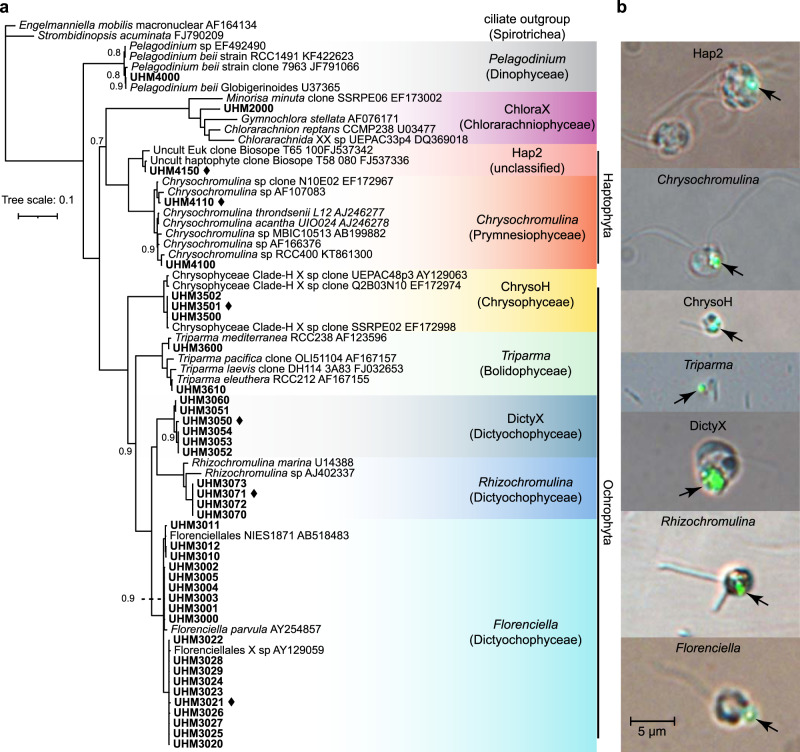
Phylogenetic diversity among the isolates and visual evidence of phagotrophy. **a** A Bayesian majority consensus tree based on near-full length 18S rRNA gene sequences, showing the phylogenetic positions of 39 mixotrophic grazers isolated from the NPSG (in bold). Genera are shaded with different colors and labeled with genus/clade and class (in parentheses). Phylum/division labels are shown vertically in cases where more than one class is represented. The six grazers for which grazing functional responses were determined are marked with black diamonds. Most branches have complete support (posterior probability = 1.0) and only those with lower support (<1.0) are labeled with support values. The scale bar indicates 10% divergence. **b** Digital micrographs of grazers consuming 0.5-µm fluorescent beads were captured using transmitted light and epifluorescence microscopy. The scale bar is 5 µm.

Diverse *Prochlorococcus*-consuming mixotrophic flagellates ranging in size from 2–5 µm were isolated from oligotrophic, open-ocean waters of the NPSG (Fig. 1, Table 1). The isolates include species from four phyla, at least six classes, and nine genera or approximately genus-level clades, referred to hereafter as genera for brevity: one *Pelagodinium* isolate in class Dinophyceae; one isolate in an undescribed clade within class Chlorarachniophyceae (hereafter referred to as ChloraX); two *Chrysochromulina* isolates and one environmental HAP-2 clade isolate (hereafter Hap2) [42] in division Haptophyta; three environmental clade H isolates in class Chrysophyceae (hereafter ChrysoH) [43]; two *Triparma* isolates in class Bolidophyceae; and twenty-nine isolates in class Dictyochophyceae. The dictyochophytes comprise nineteen *Florenciella* isolates, four *Rhizochromulina* isolates, and six isolates in an undescribed clade (hereafter DictyX). The *Florenciella* isolates cluster with two cultivated strains (*Florenciella parvula*, GenBank Acc. AY254857; Florenciellales sp. NIES 1871, GenBank Acc. AB518483) and one environmental sequence (GenBank Acc. AY129059). The three chrysophyte isolates are closely related to an environmental Chrysophyceae clade H sequence (GenBank Acc. EF172998). The closest relatives of the two *Triparma* isolates are *Triparma eleuthera* and *Triparma mediterranea*, and the two *Chrysochromulina* isolates are most closely related to an environmental *Chrysochromulina* (GenBank Acc. AF107083) and *Chrysochromulina* sp. RCC400 (GenBank Acc. KT861300), respectively. All isolates showed evidence of phagocytosis (e.g., Fig. 1b) and maintained permanent chloroplasts.

### Grazing capability and growth efficiency

**Fig. 2:**
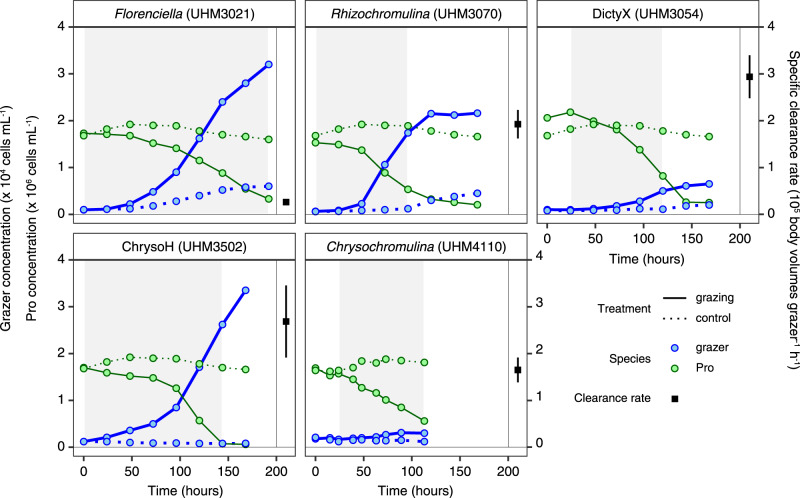
Changes in grazer and *Prochlorococcus* (Pro) prey abundance during five representative grazing experiments (results from all experiments are presented in Supplementary Fig. S1). Each panel is labeled with grazer genus/clade name and culture ID in parentheses. The square symbol on the right side of each figure shows the mean specific clearance rate (±SE) averaged over the time interval highlighted in gray. Control cultures of grazer (without prey) and prey (without grazer) are also shown in each figure.

**Fig. 3:**
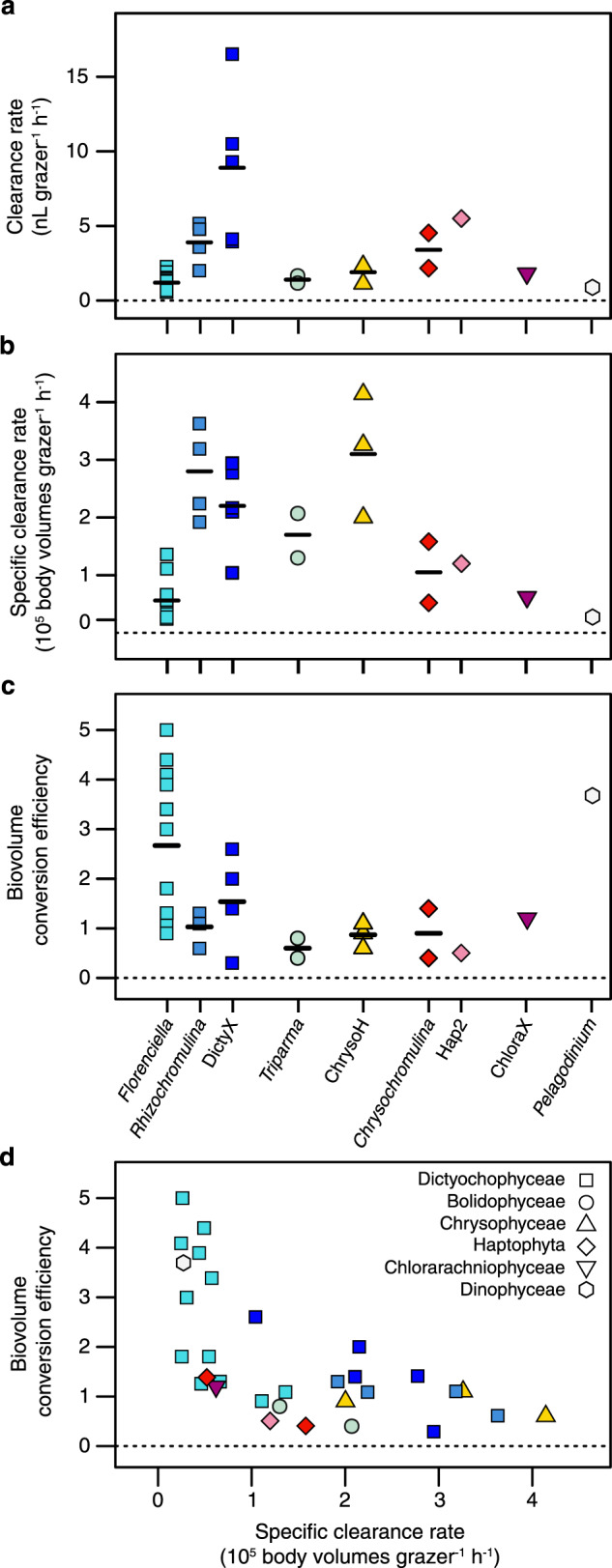
Comparison of grazing rates and growth efficiencies among NPSG mixotrophs. Panels show **a** Clearance rates or **b** specific clearance rates among isolates in different genera and biovolume conversion efficiencies for isolates **c** grouped by genus or **d** plotted as a function of specific clearance rates. Symbols in all panels are colored by genus according to labels in panels **c**, and genera within a class share a symbol shape (legend in panel **d**). For genera with multiple isolates, an arithmetic mean is shown by a horizontal bar. Note that for some isolates (marked in bold in Supplementary Table S1) replicated grazing experiments were conducted and the means are presented in this figure.

All 39 isolates were confirmed to consume *Prochlorococcus* and grew when *Prochlorococcus* was the sole added prey and primary source of nitrogen. Within a 2–8 day time course, consumption of 1–2 × 10^6^ *Prochlorococcus* mL^−1^ supported grazer growth of 10^3^–10^4^ cells mL^−1^ for the 31 different isolates (Fig. 2; Supplementary Fig. S1). Overall, grazing rates varied across phylogenetic groups, with >10-fold and ~6-fold variation in both clearance rate (volume cleared per cell) and specific clearance rate (volume cleared per body volume) across species and genera, respectively (Fig. 3a–b). Isolate UHM3050 of clade DictyX possessed the highest raw clearance rate of ~16 nL grazer^−1^ h^−1^ and strain UHM3502 of clade ChrysoH displayed the highest specific clearance rate of ~4 × 10^5^ body volumes grazer^−1^ h^−1^. On the genus level, ChrysoH also had the highest specific clearance rate (~3.1 × 10^5^ body volumes grazer^-1^ h^–1^), followed by three clades of *Rhizochromulina*, and DictyX and *Triparma* (1.7–2.8 × 10^5^ body volumes grazer^-1^ h^−1^) and *Chrysochromulina* (1.0 × 10^5^ body volumes grazer^-1^ h^−1^). *Florenciella* are among the lowest in terms of both raw and specific clearance rates (1.2 nL grazer^−1^ h^−1^, 0.6 × 10^5^ body volumes grazer^-1^ h^-1^). The remaining flagellates showed clearance rates in between these extremes, with significant variation in rates explained by genus (ANOVA on log-transformed clearance rates: *F*_8,22_ = 9.4, *p* < 10^-4^, *R*^2^ = 0.77; ANOVA on log-transformed specific clearance rates: *F*_8,22_ = 9.7, *p* < 10^-4^, *R*^2^ = 0.78).

Biovolume conversion efficiency also varied among genera, with the highest mean value observed for *Florenciella* (2.7), followed by the other two dictyochophyte clades, DictyX (1.5) and *Rhizochromulina* (1.0). Lower mean efficiencies were exhibited by *Chrysochromulina* (0.9), ChrysoH (0.9) and *Triparma* (0.6) (Fig. 3c). Strains with higher specific clearance rates tended to have lower growth efficiencies (*r* = −0.61, *p* < 10^-3^; Fig. 3d). There was substantial variation in these traits even within the Dictyochophyceae, with the highest conversion efficiencies seen in *Florenciella* strains that possessed the lowest clearance rates, and some of the lowest conversion efficiencies found in strains with higher clearance rates among *Rhizochromulina* and DictyX.

### Functional responses

**Fig. 4:**
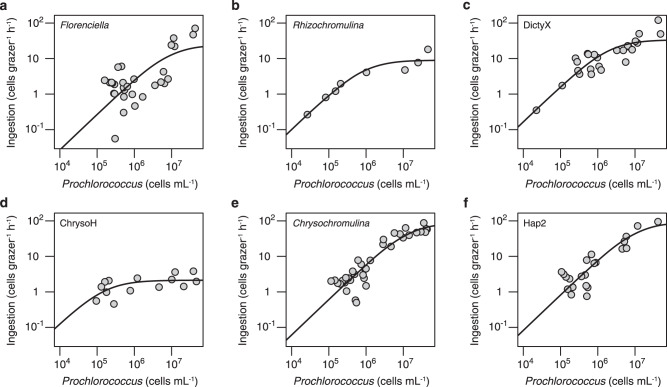
Functional responses of six representative grazers from different genera. Ingestion rates of *Prochlorococcus* are plotted as a function of prey concentration for **a** *Florenciella* (UHM3021), **b** *Rhizochromulina* (UHM3071), **c** DictyX (UHM3050), **d** ChrysoH (UHM3501), **e** *Chrysochromulina* (UHM4110), **f** Hap2 (UHM4150). Holling type II curves were fitted to the data to estimate *C*_max_ (maximum clearance rate, the initial slope of the curve) and *I*_max_ (maximum ingestion rate, the asymptote of the curve). Grazer and prey trajectories from experiments used to estimate functional responses are shown in Supplementary Fig. S6.

Functional responses were estimated for six strains representing different classes and genera (Fig. 4; strains denoted in Table 1 and Fig. 1). Functional responses displayed in terms of clearance rate instead of ingestion rate are shown in Supplementary Fig. S5. Maximum ingestion rates (*I*_max_) ranged from 2 cells grazer^−1^ h^−1^ (ChrysoH) to 92 cells grazer^−1^ h^−1^ (Hap2), with varying prey saturation concentrations between 10^5^ cells mL^−1^ for ChrysoH, 10^6^ cells mL^-1^ for *Rhizochromulina* and 10^7^ cells mL^−1^ for the remaining strains. Specific *I*_max_ (volume ingested per body volume) ranged from 0.04 body volumes grazer^−1^ h^−1^ (ChrysoH) to 0.43 body volumes grazer^−1^ h^−1^ (*Chrysochromulina*), respectively and specific *C*_max_ ranged from 0.9 × 10^5^ body volumes grazer^-1^ h^−1^ (*Florenciella*) to 1.9 × 10^6^ body volumes grazer^−1^ h^−1^ (ChrysoH) (Supplementary Table S4). Substantially higher growth of grazers and faster removal of prey were supported at higher *Prochlorococcus* concentrations (e.g., 5 × 10^6^–5 × 10^7^ cells mL^−1^), compared to rates at lower prey concentrations (<1 × 10^6^ cells mL^−1^) (Supplementary Fig. S6).

**Fig. 5:**
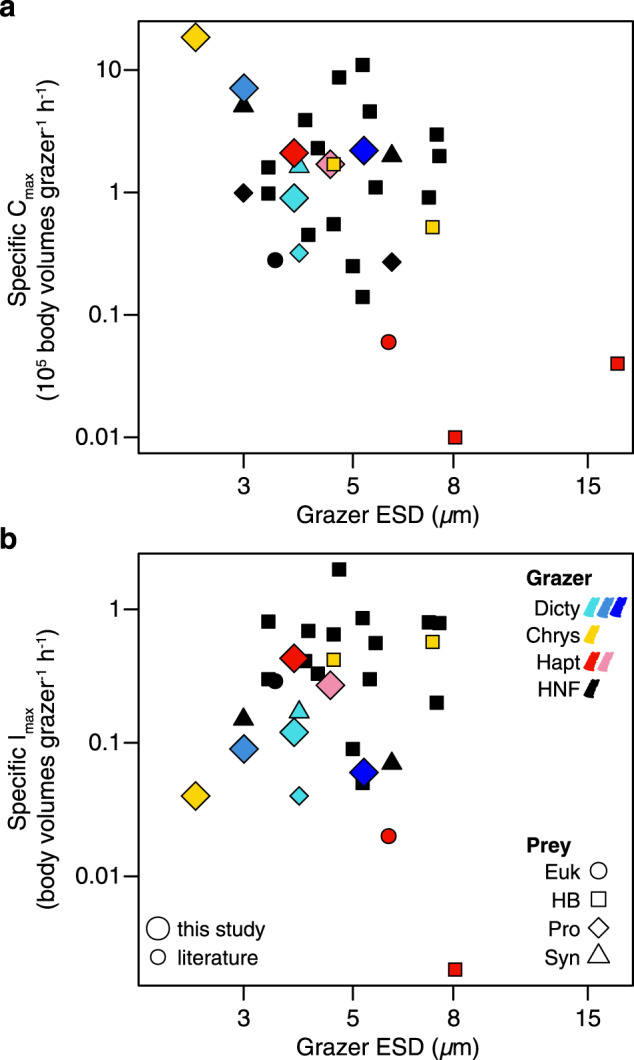
Grazing rate parameters as a function of size (equivalent spherical diameter, or ESD) for heterotrophic (HNF) and mixotrophic nanoflagellates that have been studied in culture. **a** Specific *C*_max_ (maximum volume cleared per body volume) and **b** specific *I*_max_ (maximum cell volume ingested per body volume), for flagellates grazing on *Prochlorococcus* (Pro), *Synechococcus* (Syn), heterotrophic bacteria (HB), or eukaryotic prey (Euk). In both panels (legend in **b**), black-filled symbols indicate HNF grazers, and other color-filled symbols indicate mixotrophic grazers (both from this study and literature), including chrysophytes (Chrys), dictyochophytes (Dicty) and haptophytes (Hapt). Symbol colors indicating grazer genera are the same as in Fig. 3, but shapes indicate the type of prey (source data in Supplementary Table S4). Large symbols indicate data from this study.

The body volume-specific *C*_max_ and *I*_max_ of our isolates cover nearly the whole range of values previously reported in the literature for nano-sized heterotrophic and mixotrophic flagellates, which are from <10^4^ to >10^6^ body volumes grazer^−1^ h^−1^, and <0.1 to >1 body volumes grazer^−1^ h^−1^, respectively (Fig. 5; Supplementary Table S4). It is noteworthy that the ChrysoH strain studied here, which is the smallest of our isolates (2–3 μm), demonstrated a higher specific *C*_max_ than any protistan grazer studied to date. For the one isolate previously studied and re-examined here (*Florenciella* UHM3021), grazing rates were somewhat faster than previously measured, but both prior and current *C*_max_ estimates for this strain are substantially lower than the other five isolates analyzed (Supplementary Table S4), consistent with the general pattern of low clearance rates for this genus (Fig. 3a, b).

### Abundance in the field

**Fig. 6:**
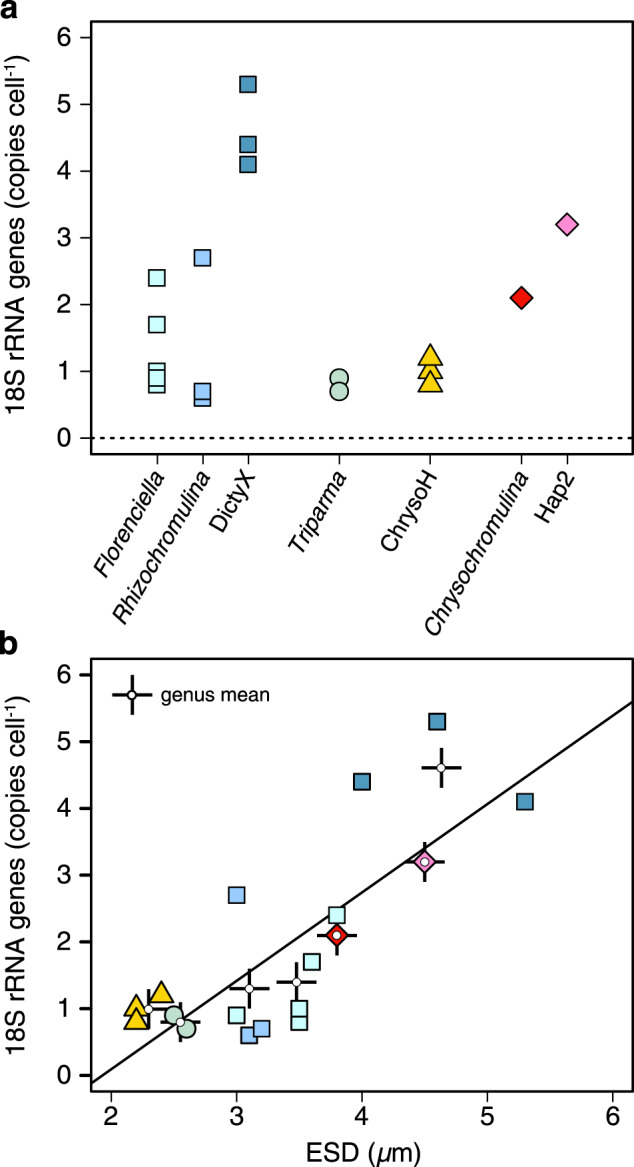
18S rRNA gene copy numbers per cell. Data are shown for **a** each of seven genera or **b** plotted as a function of equivalent spherical diameter (ESD) of the cells. Values represent the means for individual isolates (averaged over replicate measurements). In **b**, symbol colors follow the labels in **a**. Additional symbols in **b** (crosses with open circle at center) are mean values for all isolates within a genus. A least-squares linear regression line is plotted for isolate means (*R*^2^ = 0.61).

Estimates of 18S rRNA gene copy numbers indicate that all isolates tend to have low copy numbers per cell, with approximately one copy cell^−1^ for ChrysoH and *Triparma*, one to three copies cell^−1^ for *Florenciella* and *Rhizochromulina*, two copies cell^−1^ for *Chrysochromulina*, three copies cell^−1^ for Hap2, and five copies cell^−1^ for DictyX (Fig. 6a). 18S rRNA gene copy number correlated with cell size among these isolates either at the strain level (*r* = 0.78, *p* < 0.001), or when grouped by genus (*r* = 0.90, *p* < 0.01) (Fig. 6b).

**Fig. 7:**
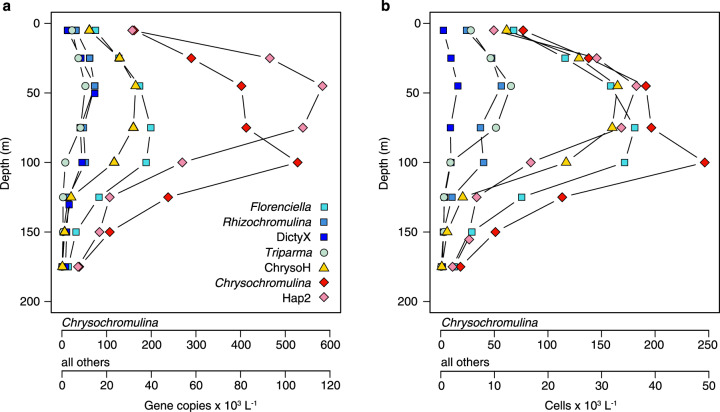
Estimated mixotroph abundances at Station ALOHA. Average in situ abundance of **a** 18S rRNA gene copies and **b** the corresponding inferred cell abundances for seven targeted clades plotted as a function of depth. Results are the averages from four depth profiles (one collected in each of four seasons during 2014). Complete source data for each season are shown in Supplementary Fig. S7.

Average abundance of 18S rRNA gene copies in the euphotic zone at Sta. ALOHA varied from 1 × 10^2^ to 5.3 × 10^5^ copies L^−1^ across groups, and estimated cell concentrations varied from 1 × 10^2^ to 2.5 × 10^5^ cells L^−1^ (Fig. 7). All groups were more abundant at upper euphotic depths (5–100 m), with abundance typically peaking at 45 m, except for *Florenciella* (75 m) and *Chrysochromulina* (100 m). *Chrysochromulina* was most abundant at every depth, peaking at 5.3 × 10^5^ copies L^−1^ (2.5 × 10^5^ cells L^−1^) at 100 m. The Hap2 clade had the second highest gene abundance (1.2 × 10^5^ copies L^−1^ at 45 m), but estimated cell abundances of Hap2, *Florenciella* and ChrysoH clades were all very similar with maxima of 3.3–3.6 × 10^4^ cells L^−1^ at 45–75 m. Lower abundances were seen for *Triparma*, *Rhizochromulina*, and DictyX clades, which had maxima ranging between 1.1–1.5 × 10^4^ copies L^−1^ (3 × 10^3^–1.3 × 10^4^ cells L^−1^) at 45 m.

Estimated contributions of each clade to the total photosynthetic picoeukaryotes in upper euphotic depths (5–100 m) were 0.1–1% (DictyX, *Rhizochromulina* and *Triparma*), 1–4% (ChrysoH, *Florenciella* and Hap2) and 9–23% (*Chrysochromulina*) (Supplementary Fig. S7a). In total these targeted clades accounted for a relative abundance between 14%–31% in upper (0–100 m) and 7–14% in lower euphotic depths (125–175 m), respectively. Group abundances varied over time (Supplementary Fig. S7b–h), with DictyX (Supplementary Fig. S7d) and Hap2 (Supplementary Fig. S7g) clades more abundant during October, while *Triparma* (Supplementary Fig. S7e) and ChrysoH (Supplementary Fig. S7f) clades were more abundant during April.

**Fig. 8:**
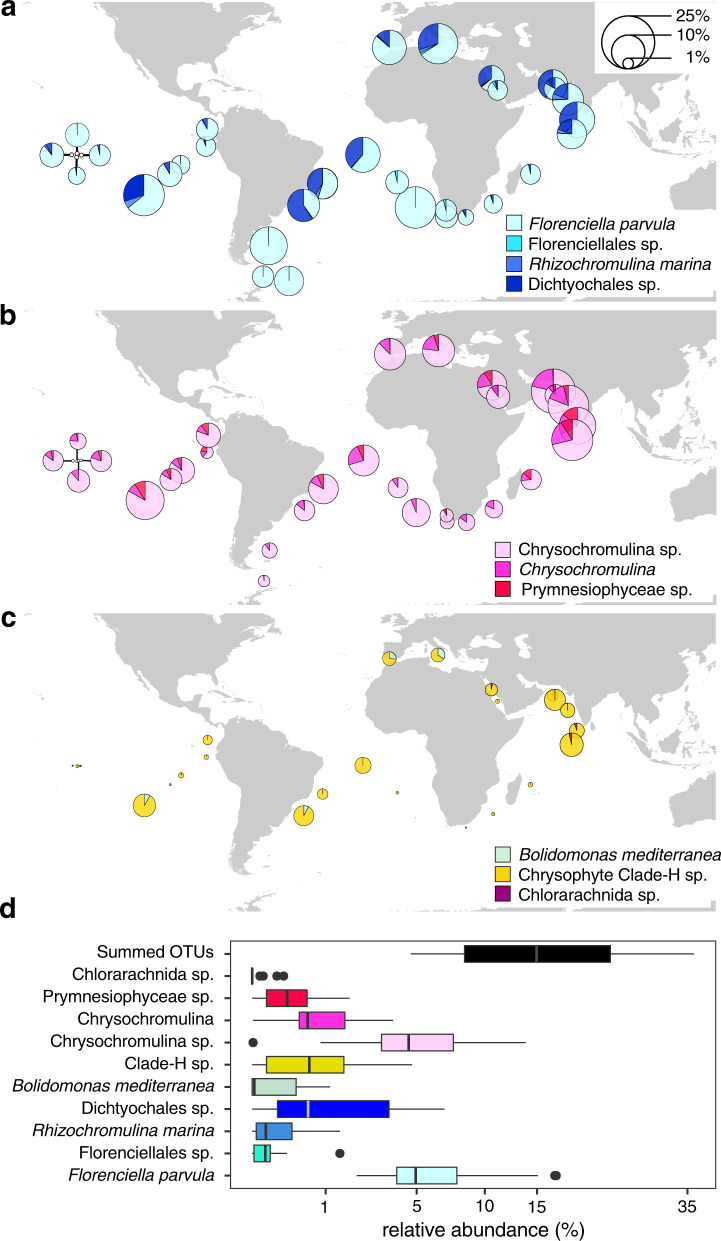
Global patterns of *Tara* Oceans OTUs closely related to our mixotroph isolates. Relative abundances of ten OTUs were grouped into class/division of **a** Dictyochophyceae, **b** Haptophyta, and **c** others, and are represented with pie charts at each station or **d** presented as box and whisker plots of the percent contributions for each targeted OTU (*n* = 40). Outliers determined by the 1.5 × IQR rule are shown as filled circles. Pie chart areas in **a**–**c** are proportional to OTU relative abundances expressed as a percentage of total phytoplankton OTUs (excluding dinoflagellates) in the 0.8–5 µm size fraction. Values in **d** are plotted on a square-root scale to improve visualization of differences between OTUs. OTUs are labeled with their lowest-level taxonomic annotations from the *Tara* Oceans dataset (full *Tara* lineages, numeric IDs, and corresponding isolates are presented in Supplementary Table S1).

Analysis of *Tara* Oceans OTUs closely related to our isolates indicates that these taxa are widespread across surface ocean samples (Fig. 8a–c), with median relative abundances ranging from <0.1% to around 5% (Fig. 8d). Dictyochophytes and haptophytes each constituted 10–25% of the community at over 10 stations, and their most abundant groups of *Florenciella parvula* and an unclassified *Chrysochromulina* sp. alone accounted for > 10% at 5 stations (Fig. 8a-b). In total the focal OTUs accounted for 5–36% (median 15%) of non-dinoflagellate phytoplankton in the 0.8–5 µm size fraction (Fig. 8d).

## Discussion

### Phylogenetically diverse and globally common mixotrophs

Marine protists are incredibly diverse and much of this diversity remains uncultured, even for the relatively well-studied phytoplankton, which makes it challenging to interpret the functional significance of environmental sequence data. The flagellates isolated and characterized in this study appear to be the only mixotrophs for which consumption of *Prochlorococcus* has been confirmed and quantified in culture-based lab experiments. Quantitative data on the grazers of *Prochlorococcus* is important because these cyanobacteria are major primary producers in oligotrophic waters and the most abundant autotroph on a global scale [13, 44]. Among the mixotrophic flagellates described here are representatives of four genera (DictyX, ChrysoH, Hap2, ChloraX) from which no cultivated representatives have previously been reported, but which appear frequently in molecular surveys. We have quantified grazing behavior in these isolates, as well as isolates from three described genera (*Rhizochromulina*, *Triparma*, and *Pelagodinium*) that had not previously been documented to consume prey. We have also studied isolates of *Chrysochromulina* from the oligotrophic open ocean, whereas previous isolates were mostly from productive coastal environments and algal bloom events.

Our isolates are smaller than most previously studied mixotrophs, perhaps reflecting their origins in the subtropical oligotrophic open ocean (Supplementary Table S5). Consistent with their small size, the estimated 18S rRNA copy numbers per cell were also low, but in line with previous estimates for picoeukaryotes using a similar approach [45]. Like the earlier study, we observed a trend of increasing copy number cell^–1^ with increasing cell size. The qPCR-based estimates in both studies are dependent on an uncertain DNA extraction efficiency and should be considered minimum estimates. However, nearly identical copy number estimates for one picoplankton, *Ostreococcus tauri*, using qPCR [45] vs draft genome assembly [46], suggest that the qPCR-based estimates are reasonable.

A search for relatives of our isolates in a global scale environmental sequencing database suggests that *Prochlorococcus*-consuming mixotrophs are common components of open-ocean ecosystems, with two genera of *Florenciella* and *Chrysochromulina* being ubiquitous in certain regions (Fig. 8a, b). The aggregate relative abundance of OTUs related to our isolates in surface *Tara* Oceans contributed up to 36% and the median abundance is similar to qPCR-based relative abundance of the targeted clades at Sta. ALOHA, i.e., 15% vs. 14% (Supplementary Fig. S7a; Fig. 8). This suggests that our isolates may represent the most abundant populations in these clades, and further efforts to estimate the absolute abundance of these globally common mixotrophs are necessary.

### A broad spectrum of trophic strategies

The NPSG is one of the most oligotrophic ecosystems in the global ocean, with persistently depleted surface nutrients and relatively stable microbial distributions [47]. The dominant eukaryotic phototrophs are small, and the 2–5 µm size class alone contributes approximately half of eukaryotic phytoplankton biomass and about one-fifth of total phytoplankton biomass [22]. This community includes non-trivial populations of haptophytes, dinoflagellates, dictyochophytes, chrysophytes, prasinophytes, bolidophytes, cryptophytes, and pelagophytes [1, 48]. Our results suggest that the high diversity of these organisms may be attributable in part to a broad spectrum of trophic strategies, including autotrophy (e.g., immotile prasinophytes) and a variety of mixotrophic strategies, illustrated by the large variation in grazing abilities among our isolates. Differences among genera in the traits we have measured suggests that the functional capacity of these communities may be somewhat predictable based on taxonomic information alone. We hypothesize that fast grazers such as the ChrysoH clade are relatively heterotrophic mixotrophs, acquiring more energy from prey and less from photosynthesis compared to slower grazers such as *Florenciella*. Although we do not have data on autotrophic capacities, a spectrum of autotrophy vs. heterotrophy is consistent with a tradeoff between clearance rate and biovolume conversion efficiency (Fig. 3d), because relatively autotrophic mixotrophs may incorporate more prey biomass into grazer biomass than relatively heterotrophic mixotrophs that respire more prey biomass for energy [49, 50]. Therefore, retention vs. remineralization of prey biomass may vary substantially across mixotrophs that co-occur in the same ecosystem, and the consequences for aggregate nutrient cycling and trophic transfer efficiency will depend on which physiologies are most abundant.

Maximum ingestion rates of the NPSG mixotrophs are intermediate or high compared to previously studied mixotrophs including haptophytes and chrysophytes, but are generally lower than most heterotrophs (Fig. 5). These results are consistent with the expectation that mixotrophs are constrained in their phagotrophic ability by tradeoffs with functions such as photosynthesis and nutrient uptake. At the same time, maximum clearance rates of the NPSG mixotrophs are intermediate-to-high compared to heterotrophs, suggesting that the two traits (*I*_max_ and *C*_max_) may be constrained by different mechanisms. *C*_max_ quantifies grazing performance when grazing is encounter-limited, and it may reflect swimming speed or feeding currents that determine encounter rate, or the efficacy of prey capture per encounter [51]. In contrast, *I*_max_ may reflect the rate of vacuole formation or other processes limiting the rate of digestion. Comparison of such traits among diverse mixotrophs, as well as phototrophic traits, would illuminate the physical basis of differences in grazing ability.

### In situ abundances and potential grazing impacts

At Station ALOHA, a typical abundance of small pigmented flagellates is ~1000–2000 cells mL^−1^ [22, 52], and many of these photosynthetic picoeukaryotes may be mixotrophic based on RNA stable isotope labeling experiments [23]. However, reports on the abundance of individual taxa of small protists are scarce [53]. Our qPCR estimates suggest that the haptophyte genus *Chrysochromulina* was the most abundant of the groups we targeted, which is consistent with its prevalence in the *Tara* Oceans data. The groups together accounted for up to ~30% of the flow cytometric counts of total photosynthetic picoeukaryotes. This may be an overestimate if gene copies per cell were underestimated. However, a recent assessment of mixotroph abundance in surface waters (5–25 m) based on ingestion of fluorescently labeled bacterial prey suggests total mixotroph concentrations of 100–200 cells mL^−1^ at the same location [17], a value only slightly lower than our combined total. The use of labeled prey may underestimate the total grazer population and our assays did not target all potential mixotrophs, so the contribution of mixotrophs to total photosynthetic eukaryotes is likely >30%. Based on a previous metabarcoding study at this location [48], the remaining small, pigmented eukaryotes likely include presumed autotrophs (immotile prasinophytes, coccolithophores, *Pelagomonas*), but also other presumed mixotrophs (dinoflagellates, cryptophytes and additional clades within the classes that our mixotrophs belong to) that we did not target (Supplementary Fig. S4).

When combining estimated clade abundances with the maximal clearance rates of our isolates (2.7–16.6 nL flagellate^−1^ h^−1^), we predict that the targeted mixotrophs could consume up to 15% of *Prochlorococcus* produced daily in upper euphotic depths (5–100 m) at Sta. ALOHA, using *Prochlorococcus* production estimates at this site from [54]. If we assume that all pigmented picoeukaryotes in this system are mixotrophs, and that they graze with a clearance rate of 6.6 nL flagellate^−1^ h^−1^ (weighted average of different mixotroph groups), then grazing mortality by mixotrophs would account for 26–50% of daily *Prochlorococcus* production in upper euphotic depths (5–100 m). These results suggest bacterial removal through mixotrophic grazing is an important process for mortality. However, the true grazing contribution from mixotrophs will depend on the relative abundance of autotrophs vs. mixotrophs, a number that remains elusive, as well as mixotroph community structure, as we have found that grazing abilities vary substantially among taxa. Furthermore, to better understand mixotrophy in ocean ecosystems it will be important to test how grazing behavior acclimates to nutrient and light availability, how the prevalence of different trophic strategies is related to environmental conditions, and whether this leads to predictable gradients in how mixotrophs affect productivity and nutrient cycling.

## Supplementary information

Supplementary Figures and Tables

## Data Availability

Primary and supplementary source data were deposited onto GitHub, including the 18S rRNA alignment, clearance rate, and growth efficiency parameters, estimates of *C*_max_ and *I*_max_ from functional response models, estimated 18S rRNA gene copies cell^−1^, qPCR depth profiles, and scripts for modeling grazing functional responses and plotting global patterns of *Tara* Oceans OTUs (https://github.com/allaboutplankton/NPSG-mixotrophs).
