# Supplementary material for: Broad phylogenetic and functional diversity among mixotrophic consumers of Prochlorococcus
Source: ISME J. 2022 Feb 10;16(6):1557–69. doi: 10.1038/s41396-022-01204-z (PMC9122939; doi:10.1038/s41396-022-01204-z)
Supplement: Supplementary file 1 — Supplementary Figures and Tables [file 41396_2022_1204_MOESM1_ESM.pdf]

Supplementary Figures and Tables
Li *et al*., Broad phylogenetic and functional diversity among mixotrophic consumers of *Prochlorococcus*

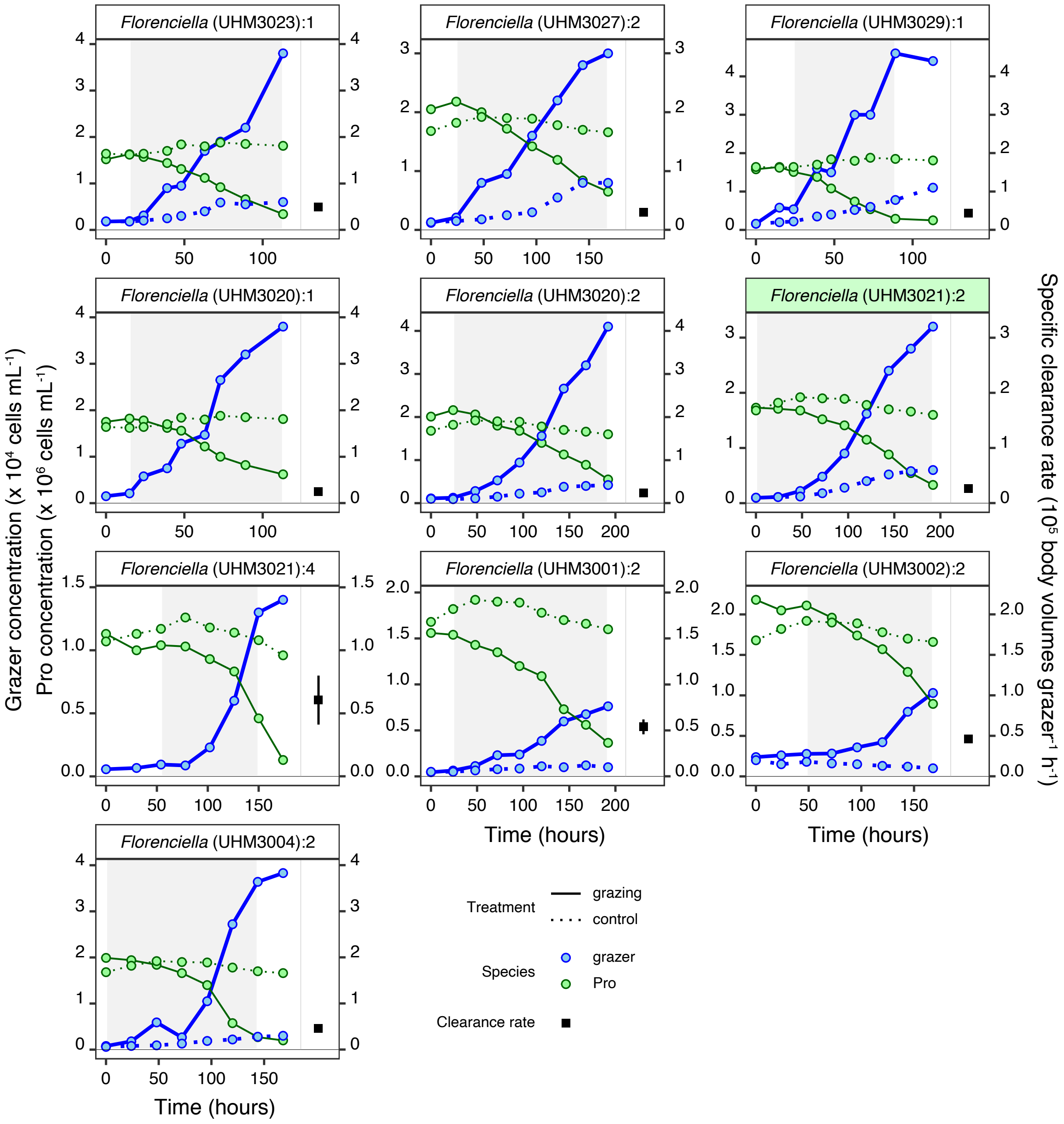

Supplementary Fig. S1. (Continued on next page)

Supplementary Figures and Tables

Li *et al*., Broad phylogenetic and functional diversity among mixotrophic consumers of *Prochlorococcus*

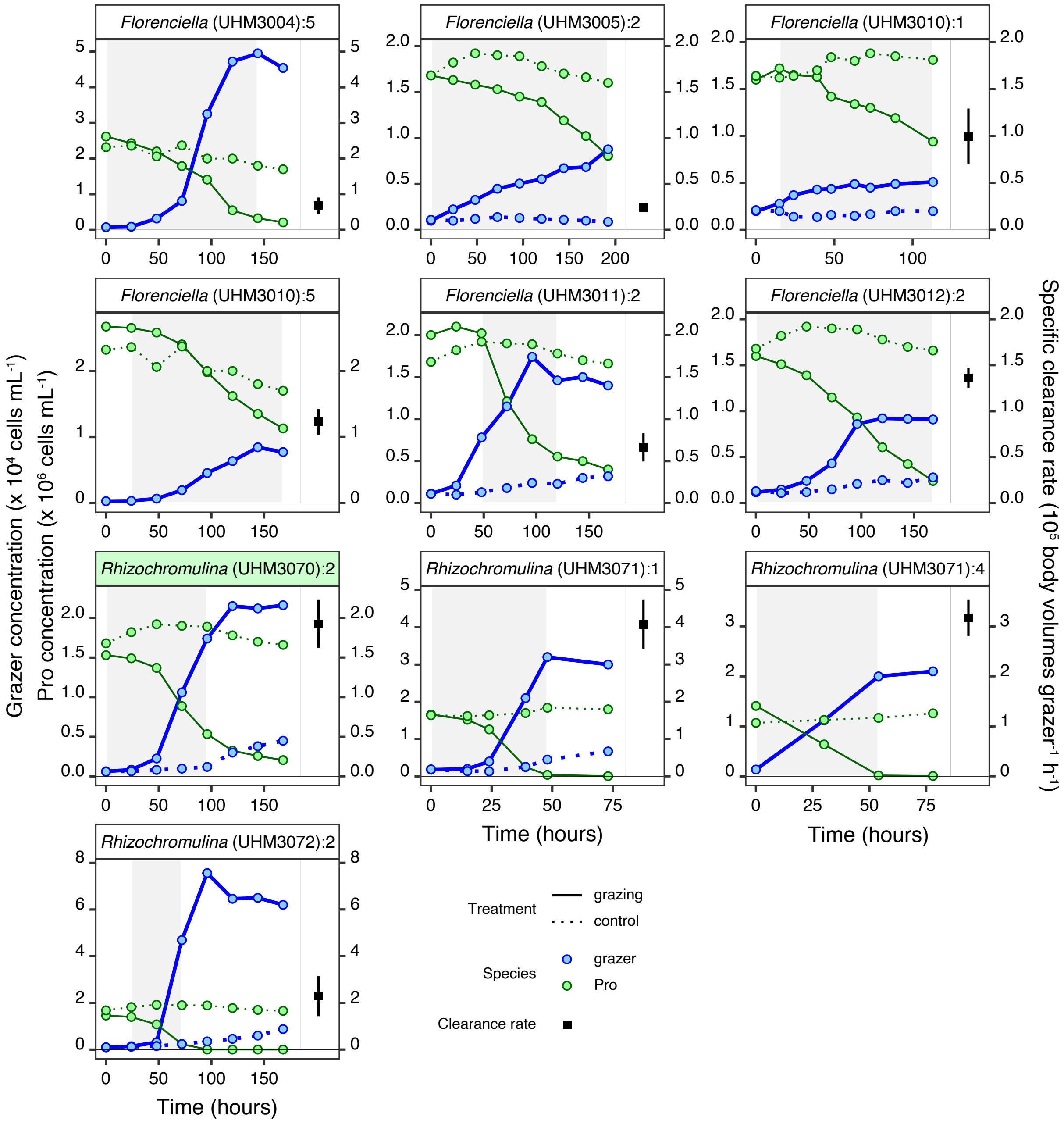

Supplementary Fig. S1. (Continued on next page)

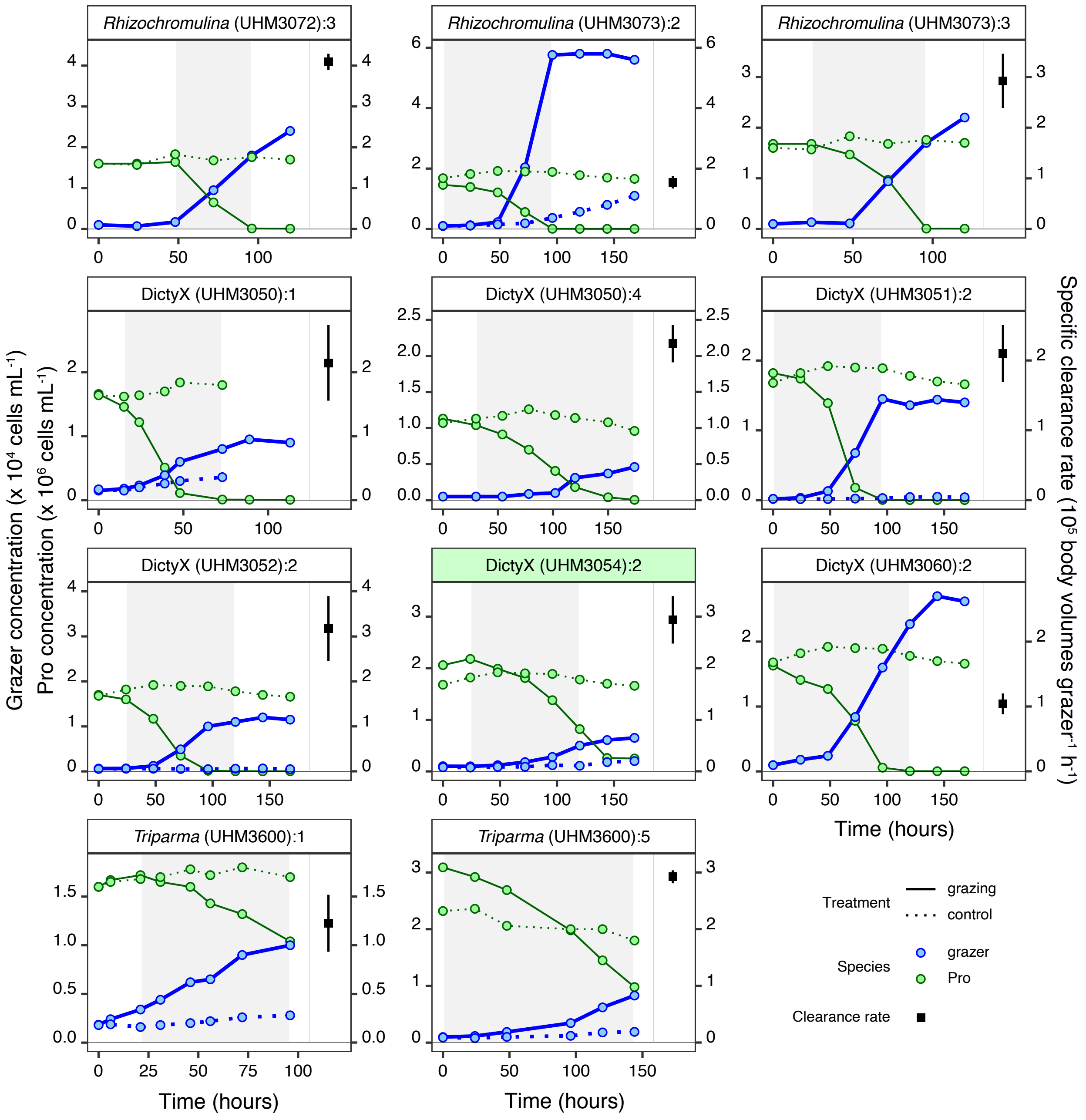

Supplementary Fig. S1. (Continued on next page)

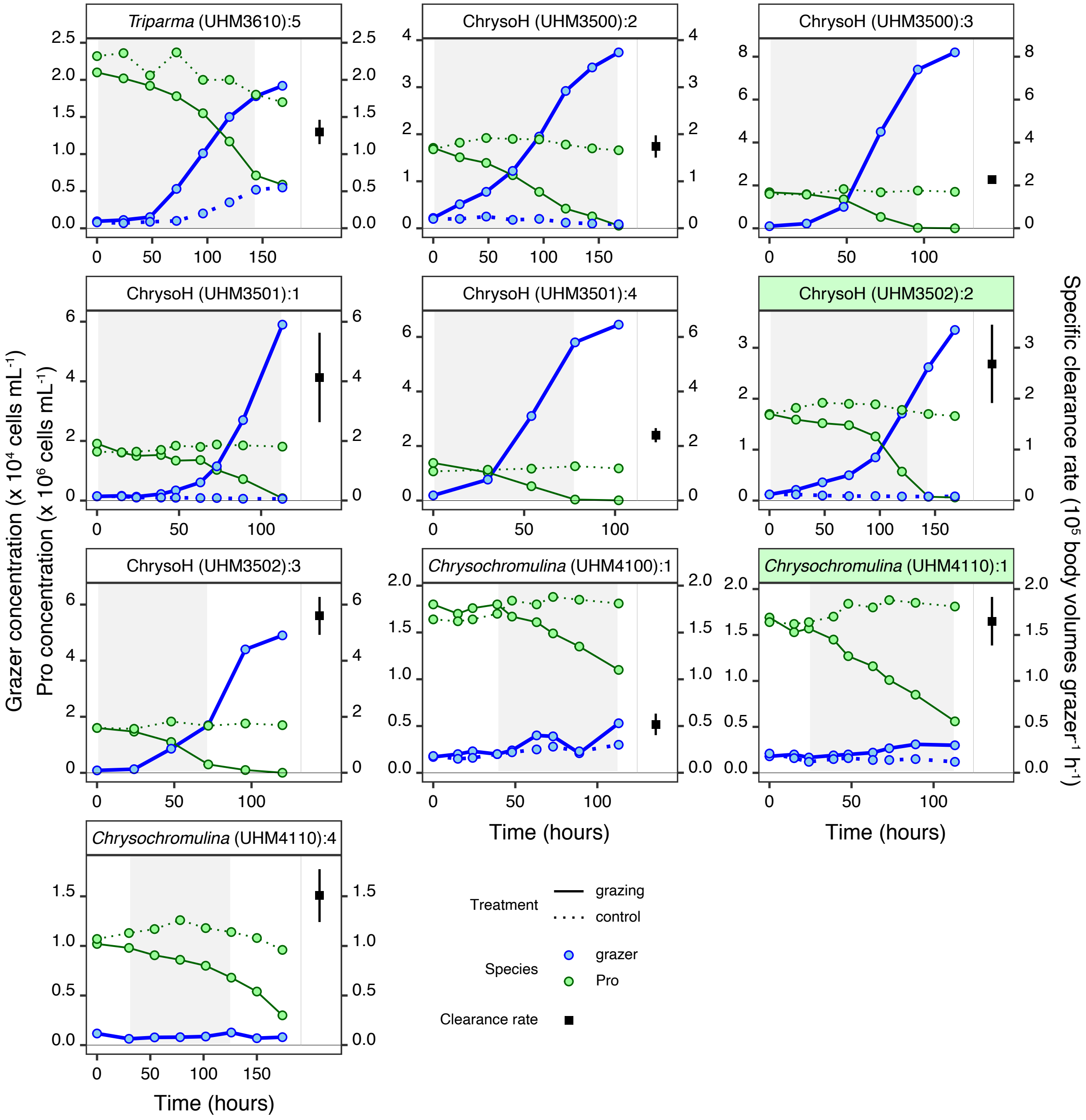

Supplementary Fig. S1. (Continued on next page)

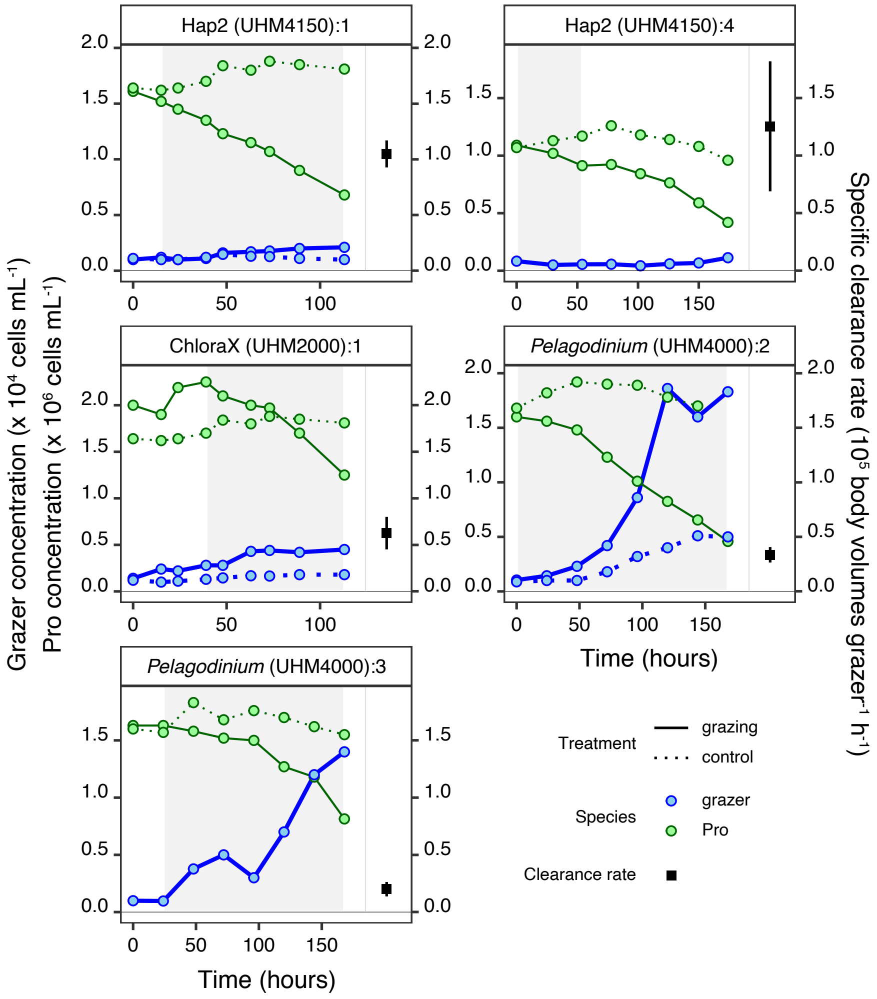

Supplementary Fig. S1. Changes in abundance of *Prochlorococcus* (Pro) prey and various grazers during experimental (prey + grazer) or control (prey without grazer or grazer without prey) incubations and the specific clearance rate (mean ± SE) derived from the data (right side of each panel). Time range highlighted in gray in each experiment indicates the data range over which specific clearance rate was calculated. Four separate rounds of experiments were conducted covering a total of 31 isolates. Fifteen of the isolates (marked in bold in Supplementary Table S1) were assayed twice, once in each of two independent rounds of experiments for a total of 46 experiments. Labels on each panel indicate: the genus or unclassified clade to which the grazer belongs, the culture identifier in parentheses, followed by a colon, and then the experimental round in which the assay occurred. Labels highlighted in green indicate the five representative experiments shown in Fig. 2 of the main text.

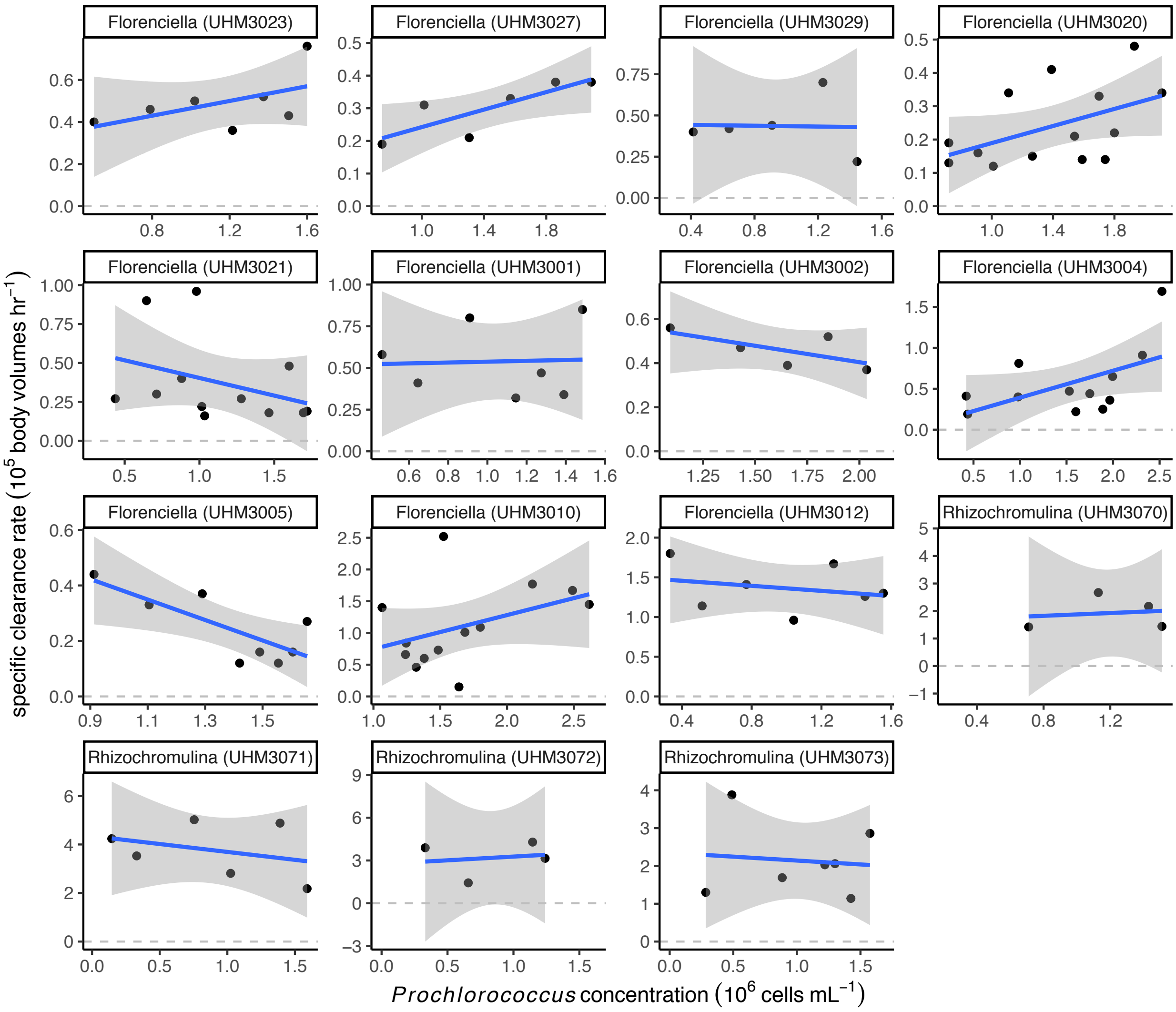

Supplementary Figure S2. (Continued on next page)

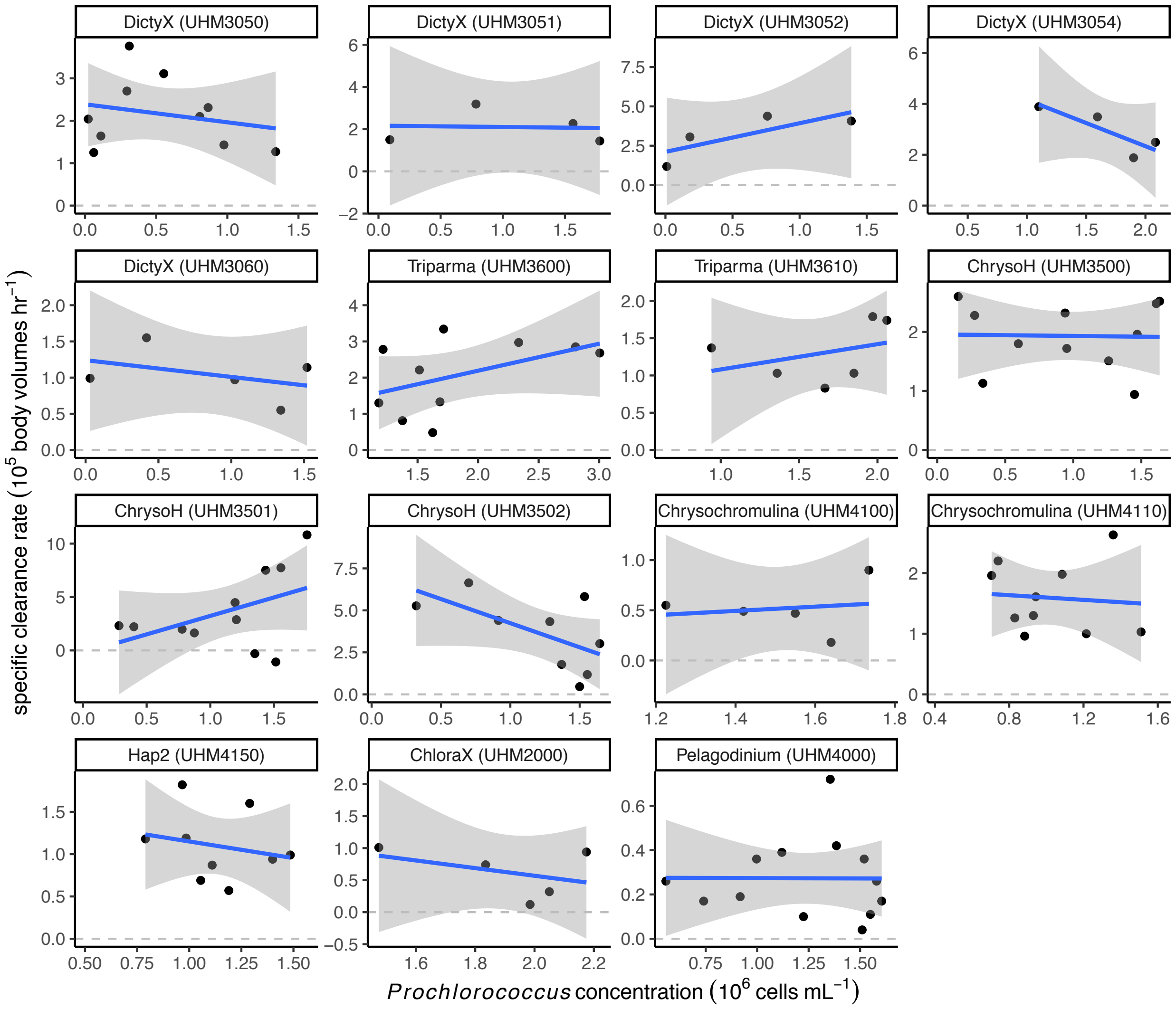

Supplementary Figure S2. Specific clearance rate as a function of *Prochlorococcus* concentration, for the experiments depicted in Fig. S1. Each panel corresponds to one isolate, with data from multiple replicates pooled. Trendlines are linear regression with 95% confidence bands. Label on each panel indicates the genus or unclassified clade to which the grazer belongs, with the culture identifier in parentheses.

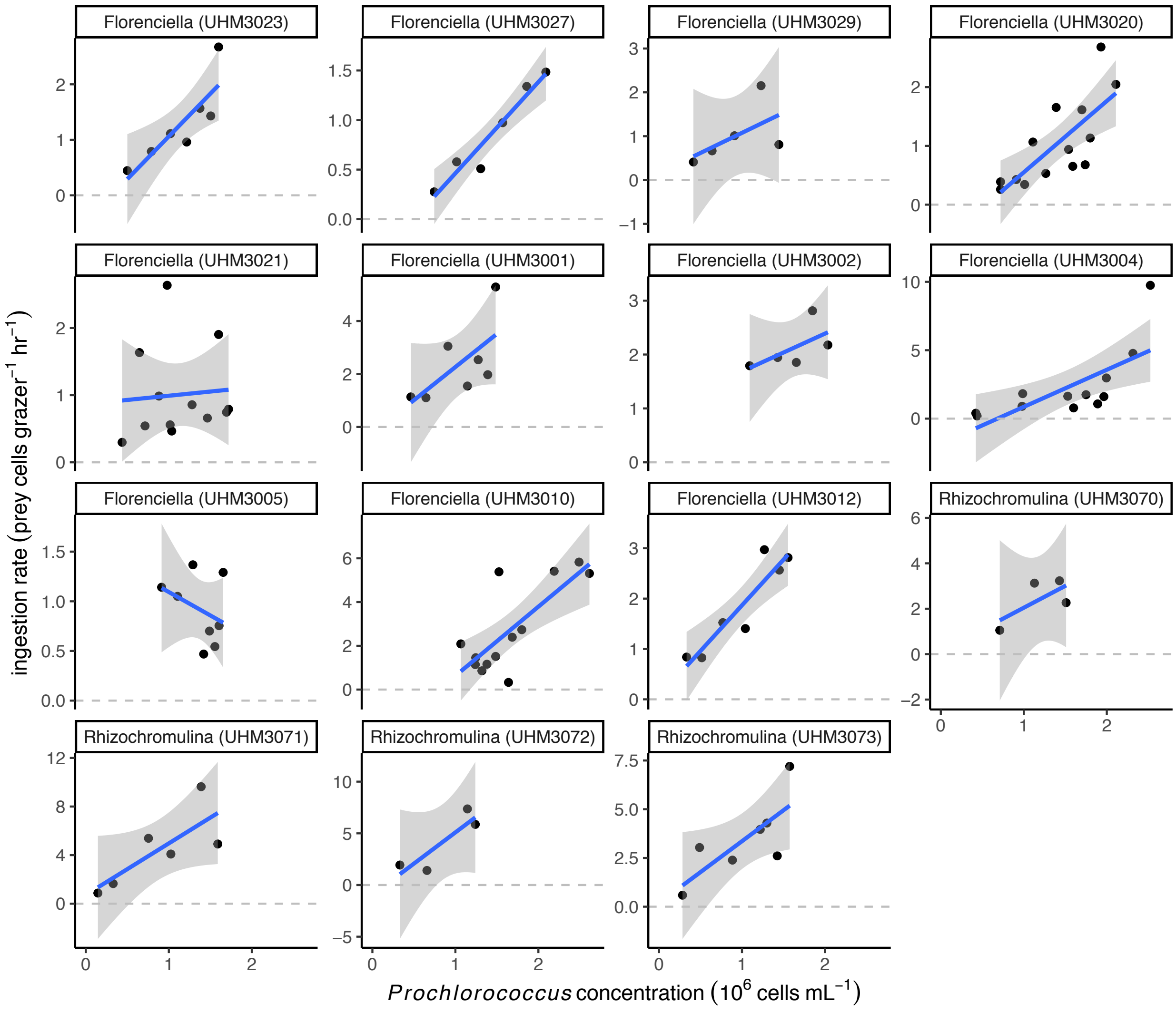

Supplementary Figure S3. (Continued on next page)

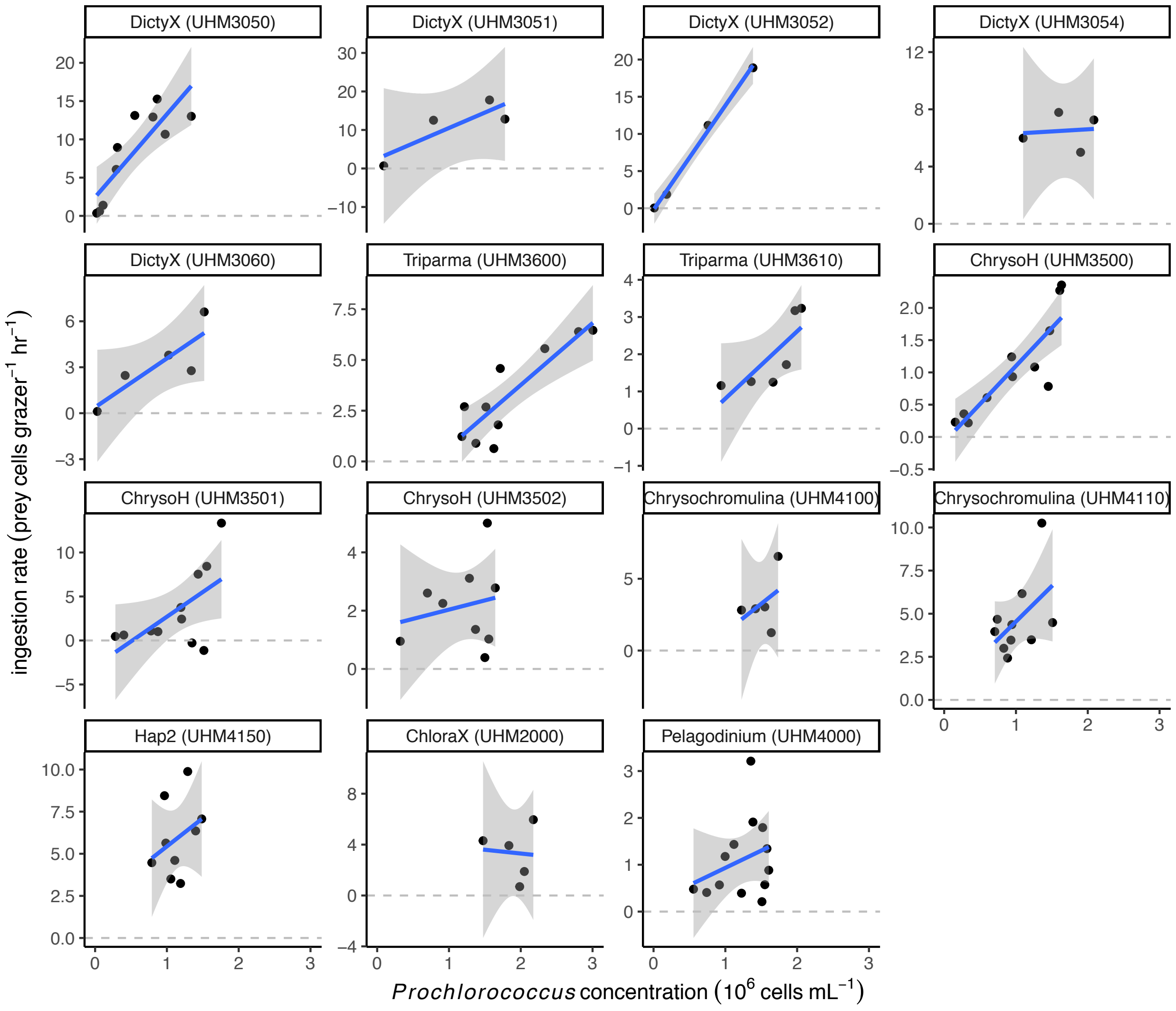

Supplementary Figure S3. Ingestion rate as a function of *Prochlorococcus* concentration, for the experiments depicted in Fig. S1. Each panel corresponds to one isolate, with data from multiple replicates pooled. Trendlines are linear regression with 95% confidence bands. Label on each panel indicates the genus or unclassified clade to which the grazer belongs, with the culture identifier in parentheses.

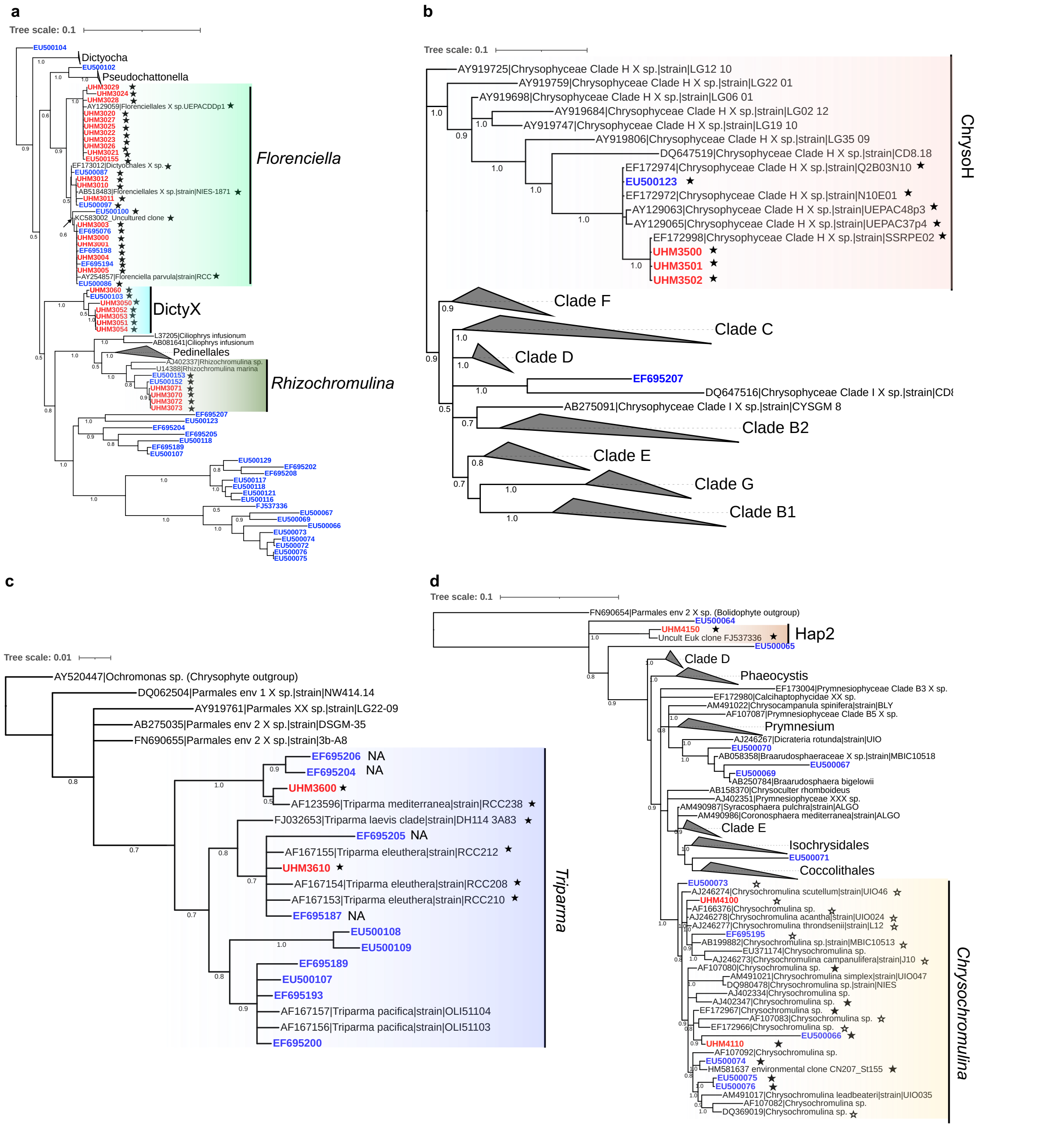

Supplementary Fig. S4. Phylogenetic diversity of the species targeted by seven qPCR primer pairs. Targeted clade indicated by labeled vertical black lines. Stars indicate OTUs within the clade having no mismatches to either primer in the pair (filled) or those with a single base pair mismatch (open). Those within the clade with no star have >1 base pair mismatch. Bayesian majority consensus trees (posterior probability shown on major branches) were inferred from partial 18S rRNA sequence alignments of our isolates (bold red), reference sequences from the PR^2 database (black), and environmental sequences of putative grazers identified by stable isotope probing [1] (bold blue) for (**a**) Dictyochophyceae, (**b**) Chrysophyceae, (**c**) Bolidophyceae and (**d**) Haptophyta. Five primer pairs (those in panels **a** and **d**) theoretically target the majority, if not all grazers in the genera (or genus-level clades). Two sets (panels b and c) target only a subset of ChrysoH or *Triparma* species/OTUs. The 'NAs' on environmental sequences within the *Triparma* clade indicate information not available because primers target a region beyond these short sequences. The scale bar indicates the fraction of divergence.

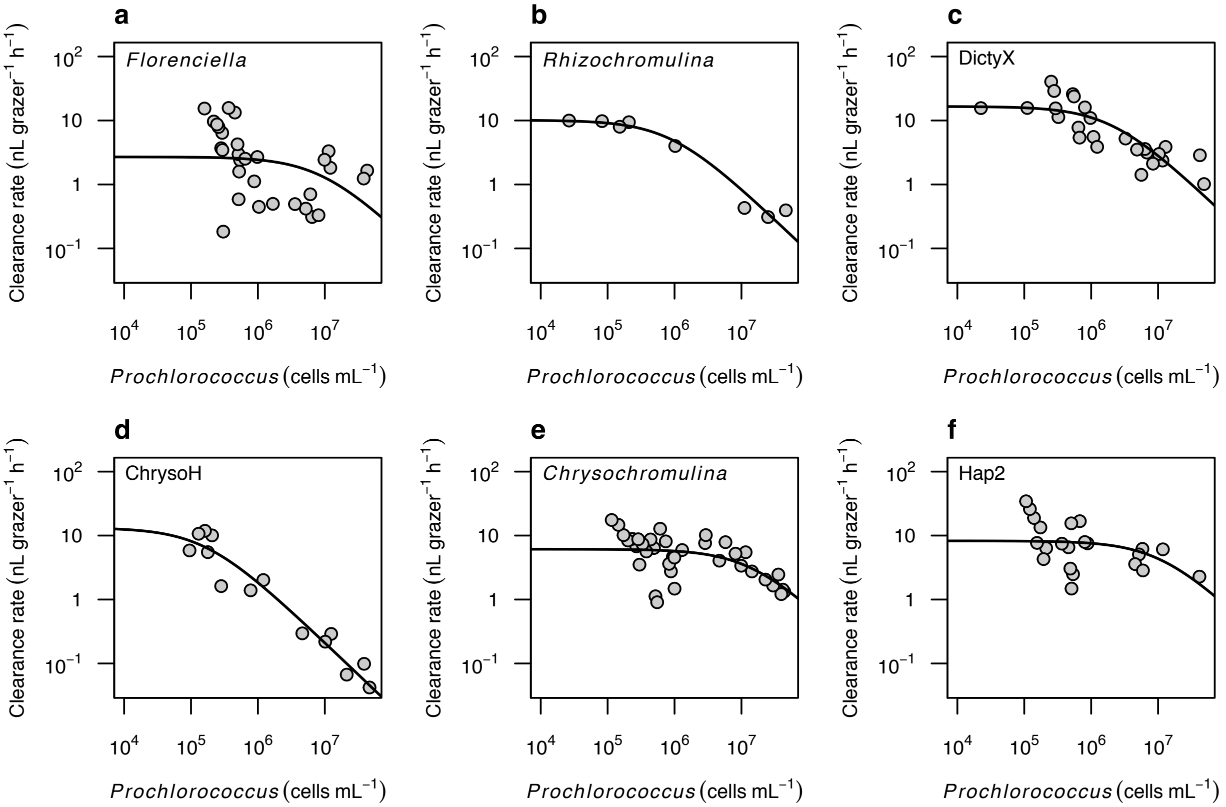

Supplementary Figure S5. Functional responses of six representative grazers from different genera, plotted with clearance rate on the y-axis rather than ingestion rate. Clearance rates of *Prochlorococcus* are plotted as a function of prey concentration. Holling type II curves were fitted to the data to estimate C_{max} (maximum clearance rate, the initial slope of the curve) and I_{max} (maximum ingestion rate, the asymptote of the curve). Grazer and prey trajectories from experiments used to estimate functional responses are shown in Supplementary Fig. S6.

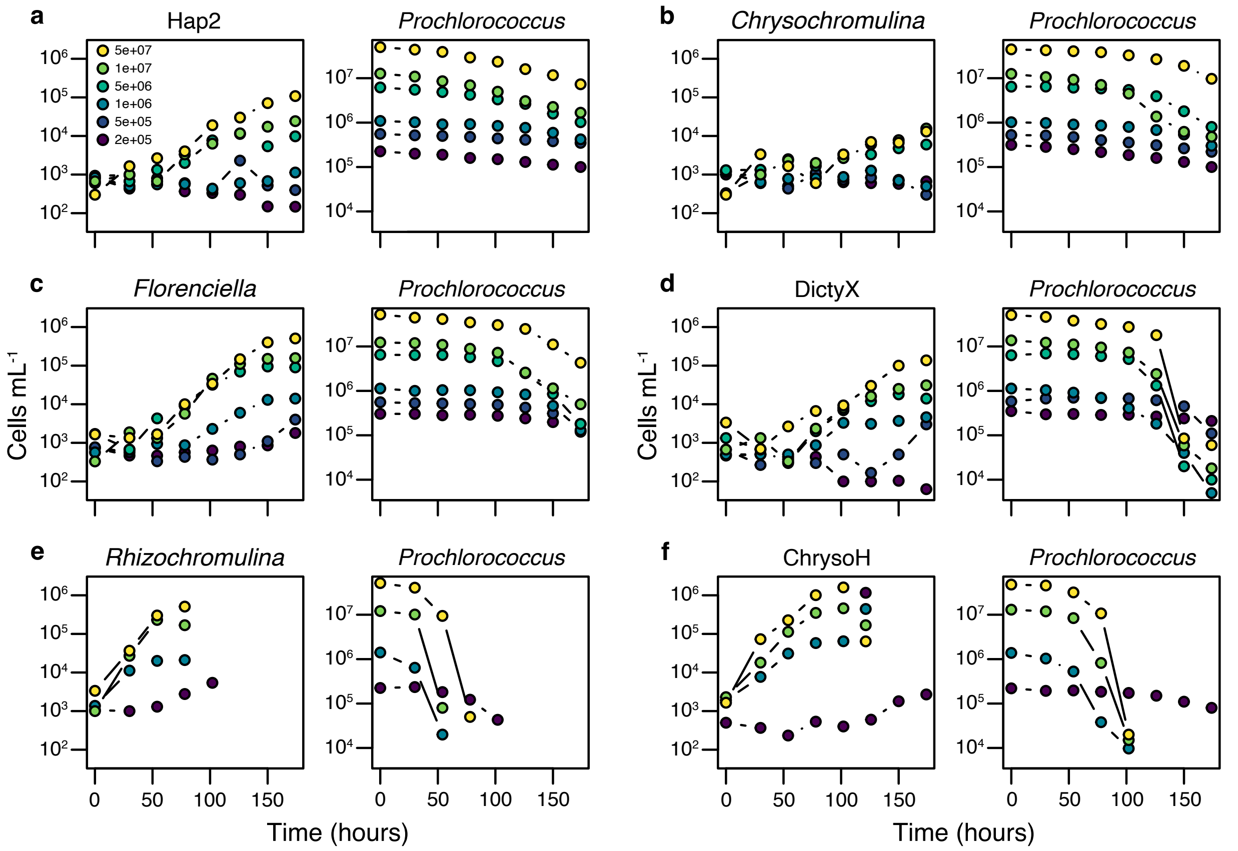

Supplementary Fig. S6. Variation in grazer and prey trajectories (adjacent pairs of panels) during grazing experiments with six isolates investigated for functional responses. Specific grazer strain identifiers are (**a**) Hap2 (UHM4150), (**b**) *Chrysochromulina* (UHM4110), (**c**) *Florenciella* (UHM3021), (**d**) DictyX (UHM3050), (**e**) *Rhizochromulina* (UHM3071), and (**f**) ChrysoH (UHM3501). Symbol colors indicate the concentration of added *Prochlorococcus* (cells mL^{-1}) as shown in the legend in panel **a**.

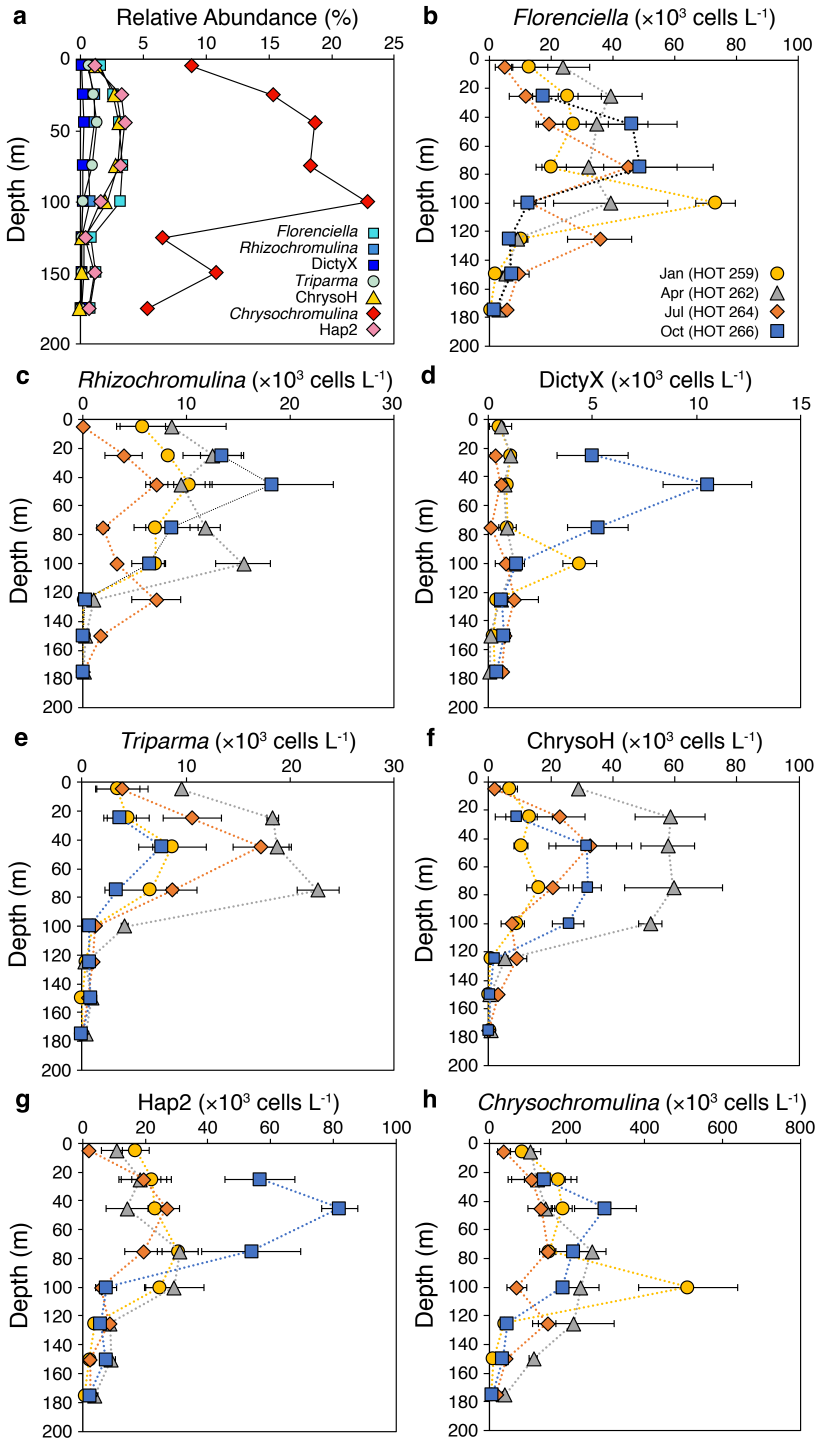

Supplementary Figure S7. Relative and absolute abundances of seven mixotroph groups as a function of depth in the euphotic zone. (**a**) Annual average abundance of each group expressed as percentage of total pigmented eukaryotes at the same depths and times. (**b–h**) Variations in the abundance of seven mixotrophic groups collected in four different seasons at Station ALOHA during Hawaii Ocean Time-series (HOT) cruises 259, 262, 264 and 266, which took place in Jan, Apr, Jul and Oct of 2014, respectively. Total pigmented picoeukaryote counts were acquired from the Hawai‘i Ocean Time Series Data Organization and Graphical System: https://hahana.soest.hawaii.edu/hot/hot-dogs/).

Supplementary Table S1. Taxonomy, culture IDs, and comparable *Tara* Oceans OTU information for isolates described in this study.

Phylum|Class	Genus or Clade[1]	Species[2]	Strain[3]	Culture ID[4]	*Tara* lineage[5]	*Tara* ID[6]
**Ochrophyta**						
Dictyochophyceae	*Florenciella*	*F. parvula*	PX-310-100-01	*UHM3000*	Eukaryota|Stramenopiles|Stramenopiles_X|Dictyochophyceae|Dictyochophyceae_X|Florenciellales|Florenciella|Florenciella+parvula	146146
			PX-310-100-02	UHM3001		
			PX-310-100-03	UHM3002		
			PX-310-100-04	*UHM3003*		
			**PX-310-100-05**	**UHM3004**		
			PX-310-100-07	UHM3005		
			**PX-311-25-03**	**UHM3010**		
			PX-311-100-01	UHM3011		
			PX-311-100-03	UHM3012		
		sp.	**AL-45-004C**	**UHM3020**	Eukaryota|Stramenopiles|Stramenopiles_X|Dictyochophyceae|Dictyochophyceae_X|Florenciellales| Florenciellales_X|Florenciellales X+sp.	73377
			**UA-265-01**	**UHM3021**		
			PX-310-25-01	UHM3022		
			PX-310-25-04	UHM3023		
			PX-310-25-05	*UHM3024*		
			PX-310-75-01	*UHM3025*		
			PX-310-75-02	*UHM3026*		
			PX-310-75-03	UHM3027		
			PX-310-75-04	*UHM3028*		
			PX-310-75-05	UHM3029		
	Rhizochromulina	sp.	PX-311-25-09	UHM3070	Eukaryota|Stramenopiles|Stramenopiles_X|Dictyochophyceae|Dictyochophyceae_X|Rhizochromulinales|Rhizochromulina|Rhizochromulina+marina	67389
			**PX-311-75-01**	**UHM3071**		
			**PX-311-75-04**	UHM3072		
			**PX-311-75-05**	UHM3073		
	Dictyochophyceae-X (DictyX)	sp.	**UA-269-03**	**UHM3050**	Eukaryota|Stramenopiles|Stramenopiles_X|Dictyochophyceae|Dictyochophyceae_X|Dictyochales|Dictyochales_X|Dictyochales_X+sp.	46831
			PX-310-25-02	UHM3051		
			PX-310-25-03	UHM3052		
			PX-311-25-08	*UHM3053*		
			PX-311-100-05	UHM3054		
			PX-311-25-02	UHM3060		

Supplementary Table S1. (Continued)

<table>
<tr><th></th><th></th><th></th><th></th><th></th><th></th><th></th></tr>
<tr><td>Chrysophyceae</td><td>Chrysophyceae-H (ChrysoH)</td><td>sp.</td><td>PX-311-25-01</td><td>UHM3500</td><td>Eukaryota|Stramenopiles|Stramenopiles_X|Chrysophyceae-Synurophyceae|Chrysophyceae-Synurophyceae_X|Clade-H|Clade-H_X|Clade-H_X+sp.</td><td>115485</td></tr>
<tr><td></td><td></td><td></td><td>PX-311-25-04</td><td>UHM3501</td><td></td><td></td></tr>
<tr><td></td><td></td><td></td><td>PX-311-25-05</td><td>UHM3502</td><td></td><td></td></tr>
<tr><td>Bolidophyceae</td><td>Triparma</td><td>T. mediterranea</td><td>PX-311-25-07</td><td>UHM3600</td><td>Eukaryota|Stramenopiles|Stramenopiles_X|Bolidophyceae-and-relatives|Bolidophyceae-and-relatives_X|Bolidophyceae-and-relatives_XX|Bolidomonas|Bolidomonas+mediterranea</td><td>239078</td></tr>
<tr><td></td><td></td><td>T. eleuthera</td><td>PX-311-100-02</td><td>UHM3610</td><td></td><td></td></tr>
<tr><td>Myzozoa[7]</td><td></td><td></td><td></td><td></td><td></td><td></td></tr>
<tr><td>Dinophyceae</td><td>Pelagodinium</td><td>P. beii</td><td>PX-311-25-11</td><td>UHM4000</td><td>—</td><td>—</td></tr>
<tr><td>Chlorarachniophyceae</td><td>Chlorarachniophyceae-X (ChloraX)</td><td>sp.</td><td>AL-FL05</td><td>UHM2000</td><td>Eukaryota|Rhizaria|Cercozoa|Filosa-Chlorarachnea|Chlorarachnida|Chlorarachnida_X</td><td>174670</td></tr>
<tr><td>Prymnesiophyceae</td><td>Chrysochromulina</td><td>sp.</td><td>NF-H275-5m<3</td><td>UHM4110</td><td>Eukaryota|Hacrobia|Haptophyta|Prymnesiophyceae|Prymnesiales|Chrysochromulinaceae|Chrysochromulina| Chrysochromulina_X+sp.</td><td>178099</td></tr>
<tr><td></td><td></td><td></td><td>AL-TEMP-12</td><td>UHM4100</td><td>Eukaryota|Hacrobia|Haptophyta|Prymnesiophyceae|Prymnesiales|Chrysochromulinaceae|Chrysochromulina</td><td>11632</td></tr>
<tr><td></td><td>Haptophyta-2 (Hap2)</td><td>sp.</td><td>PX-310-25-06</td><td>UHM4150</td><td>Eukaryota|Hacrobia|Haptophyta|Prymnesiophyceae|Prymnesiophyceae_X|Prymnesiophyceae_XX|Prymnesiophyceae_XXX|Prymnesiophyceae_XXX+sp.</td><td>162435</td></tr>
</table>

[1] DictyX, ChrysoH, ChloraX, and Hap2 are abbreviations for undescribed or environmental approximately genus-level clades of Dictyochophyceae, Haptophyta, Chrysophyceae, and Chlorarachniophyceae, respectively.

[2] Species assignments are putative and based on high similarity of the near-full length 18S rRNA gene phylogeny of the isolate and known species

[3] 'Strain' codes are the original unique identifiers used at the time of isolation.

[4] Culture IDs are identifiers assigned to cultures of protists isolated by our group at University of Hawai'i at Mānoa (UHM####) that are reported in publications. Grazing rates were measured at least once on all isolates and were measured twice in two independent experiments for the fifteen isolates in bold font. Culture IDs in grey italic font were found to graze on *Prochlorococcus*, but rates were not calculated, because of low sampling resolution.

[5] '*Tara* lineage' is the taxonomic annotation of the most similar OTU in the *Tara* dataset (E-value $<10^{-15}$).

[6] '*Tara* ID' lists the identifier of the 18S rRNA V9 reference sequence of the OTU http://taraoceans.sb-roscoff.fr/EukDiv/

[7] '*Tara* lineage and ID not shown, because dinoflagellates were not included in the analysis of global distribution of mixotrophs as explained in the main text

Supplementary Table S2. Detailed information of group-specific qPCR primers designed for targeting various mixotrophs in situ.

**Genus or Clade[1]**	**Primer**	**Sequence (5'–3')**	**T_m (℃)**	**Amplicon length (bp)**	**qPCR efficiency**
Florenciella	Florenciella-652F	GGA TTT CTG GTG GGG CTG TC	58	132	1.06
	Florenciella-784R	GTA AAC GAC GAG CGT CAC TCC	58		
Rhizochromulina	Rhizochromulina-700F	GTG TTG TCT CCG GTT CTA TTT CG	56	176	0.99
	Rhizochromulina-876R	GGC AGA ACC ACC AAA GTC CT	58		
Dictyophyceae-X (DictyX)	DictyX-1638F	CGT CTT TCG TGA TAG GGA TAG	52	165	0.99
	DictyX-1803R	AAG CGG TAC TCC TCA TGT C	54		
Chrysochromulina	Chrysochromulina-291F	TGC CGA TGG GTA CGC ACT G	60	95	0.96
	Chrysochromulina-386R	AGT AAA CGT TCC GTC TCC CGC C	61		
Haptophyta-2 (Hap2)	Hap2-291F	TGC CGA TGG GTA TGC ACT G	58	95	0.99
	Hap2-386R	AGT AAA AGT CCT GTT TCC TAC GCC	57		
Chrysophyceae-H (ChrysoH)	ChrysoH-694F	CAC CTC ACG GTG TCT GTA C	55	108	0.99
	ChrysoH-802R	AAT GAC GAG ATT CCC RAG C	54		
Triparma	Triparma-1632F	AAC CTT GAC CGA GAG GTC TG	56	122	0.95
	Triparma-1754R	CAA TGC AGT CTG ATG AAC TGC	54		

[1] DictyX, Hap2, and ChrysoH are abbreviations for unclassified genus-level clades in Dictyochophyceae, Haptophyta, and Chrysophyceae, respectively

Supplementary Table S3. Mismatches between primers and target sites in representative isolates and empirical tests of primer specificity as determined by PCR amplification success.

Primer Pair / Isolate	*Florenciella* 652F/784R		*Rhizochromulina* 700F/876R		DictyX 1638F/1803R		*Triparma* 1632F/1754R		ChrysoH 694F/802R		*Chrysochromulina* 291F/386R		Hap2 291F/386R	
	Mis-match[1]	Band[2]	Mis-match	Band	Mis-match	Band	Mis-match	Band	Mis-match	Band	Mis-match	Band	Mis-match	Band
Florenciella parvula	0	+	17	-	11	-	9		16		20	-	23	
Florenciella sp.	0	+	17	-	11	-	7		17		17	-	21	
Rhizochromulina sp.	10	-	0	+	10	-	10		15		14	-	21	
DictyX sp.	8	-	21	-	0	+	10		21		22	-	23	
Triparma mediterranea	16	-	25		17		0	+	23	-	29		24	
Triparma eleuthera	15		25		14		0	+	23	-	29		28	
ChrysoH sp.	19		24		12		9	-	0	+	26	-	25	-
Chrysochromulina sp.[3]	17		20		19		9	-	17	-	0	+	9	-
Chrysochromulina sp.[4]	16		20		19		9	-	17	-	1	-	9	-
Hap2 sp.	19		20		20		9	-	17	-	9	-	0	+

[1]Number of base pair mismatches between each primer pair and target sites in representative isolates (listed by genus and species or clade name) as determined after alignment using the Geneious software (http://www.geneious.com).

[2]Primer specificity was tested by PCR using both target (positive control) and non-target (negative control) isolate DNA as template followed by agarose gel electrophoresis to determine presence or absence of a band. Successful amplifications are indicated by green shaded cells with '+', and amplifications for which no band was visible are shown as gray shaded cells with "-". Combinations which were not empirically tested have no shading.

[3]*Chrysochromulina* strain NF-N275-5m<3, Culture ID UHM4110.

[4]*Chrysochromulina* strain AL-TEMP-12, Culture ID UHM4100.

Supplementary Table S4. Maximum clearance rate and maximum ingestion rate parameters for heterotrophic and mixotrophic nanoflagellates†.

Genus or Clade	Species or Culture ID	V	I_{max}	C_{max}	Nutrition	Prey	Reference
Chrysochromulina	*ericina*	108	0.02	0.06	mix	euk	[2]
Chrysochromulina	*polylepis*	2680	NA	0.04	mix	hb	[3]
Paraphysomonas	sp.	113	NA	0.27	het	pro	[4]
Paraphysomonas	sp.	113	0.07	1.99	het	syn	[4]
Prymnesium	*patellliferum*	275	0	0.01	mix	hb	[5]
Pseudobodo	sp.	14	NA	0.99	het	pro	[6]
Pseudobodo	sp.	14	0.15	5.08	het	syn	[6]
Actinomonas	*mirabilis*	75	0.86	11.0	het	hb	[7]
Bodo	*designis*	54	1.99	8.70	het	hb	[7]
Ciliophrys	*infusionum*	220	0.79	1.98	het	hb	[7]
Codosiga	*gracilis*	35	0.69	0.45	het	hb	[7]
Diaphanoeca	*grandis*	40	0.33	2.30	het	hb	[7]
Jakoba	*libera*	75	0.05	0.14	het	hb	[7]
Monosiga	sp.	20	0.81	0.98	het	hb	[7]
Ochromonas	sp.	50	0.42	1.70	mix	hb	[7]
Ochromonas	sp.	200	0.57	0.52	mix	hb	[7]
Paraphysomonas	*imperforata*	212	0.2	2.97	het	hb	[7]
Paraphysomonas	*vestita*	190	0.8	0.91	het	hb	[7]
Pleoromonas	*jaculans*	50	0.65	0.55	het	hb	[7]
Pseudobodo	*tremulans*	90	0.56	1.10	het	hb	[7]
Pseudobodo	sp.	34	0.41	3.90	het	hb	[7]
Pseudobodo	sp.	22	0.29	0.28	het	euk	[7]
Spurnella	sp.	65	0.09	0.25	het	hb	[7]
Stephanoeca	*diplocostata*	20	0.30	1.60	het	hb	[7]
Stephanoeca	*diplocostata*	83	0.30	4.58	het	hb	[7]
Florenciella	UHM3021	29	0.04	0.32	mix	pro	[8]
Florenciella	UHM3021	29	0.17	1.62	mix	syn	[8]
Florenciella	**UHM3021**	**29**	**0.12**	**0.90**	**mix**	**pro**	**this study**
Rhizochromulina	**UHM3071**	**14**	**0.09**	**7.10**	**mix**	**pro**	**this study**
**DictyX**	**UHM3050**	**76**	**0.06**	**2.20**	**mix**	**pro**	**this study**
Chrysochromulina	**UHM4110**	**29**	**0.43**	**2.09**	**mix**	**pro**	**this study**
**Hap2**	**UHM4150**	**48**	**0.27**	**1.70**	**mix**	**pro**	**this study**
**ChrysoH**	**UHM3501**	**7**	**0.04**	**18.5**	**mix**	**pro**	**this study**

†Values were collected from published studies and this study (in bold). Volume (V) = grazer body volume in μm^3, I_{max} = specific maximum ingestion rate (body volumes consumed grazer^{-1} h^{-1}), C_{max} = specific maximum clearance rate (×10^5 body volumes cleared grazer^{-1} h^{-1}), nutrition = mixotroph (mix) or heterotroph (het), prey = heterotrophic bacteria (hb) or *Prochlorococcus* (pro) or *Synechococcus* (syn) or eukaryotic alga (euk). In some cases, C_{max} and I_{max} were not reported in the original study, and we estimated them by fitting a type II functional response to reported data.

Supplementary Table S5. Nano-sized, non-dinoflagellate marine eukaryotes reported to have phago-mixotrophic capability†.

Phylum|Class	Genus or Clade	Size (µm)	Primary evidence of mixotrophy	Original publication
**Chlorophyta**				
Mamiellophyceae	*Dolichomastix*	~5	ingestion of live bacteria	[12]
	Mantoniella	<5	ingestion of microspheres or FLB	[13][14]
Nephrophyceae	*Nephroselmis*	~5	ingestion of live bacteria	[10][12]
Pyramimonadophyceae	*Cymbomonas*	>10	ingestion of live bacteria	[15][12]
	Pyramimonas	>10	ingestion of FLB, microspheres or live bacteria	e.g. [12][13][16]
**Ochrophyta**				
Bolidophyceae	*Triparma*	~3	consumption and growth on *Prochlorococcus*	**this study**
Chrysophyceae	*Ochromonas*	>5	ingestion of bacteria or FLB	e.g. [17][18]
	Dinobryon	>3	ingestion of latex-beads or FLB	[19][20]
	ChrysoH	~2	consumption and growth on *Prochlorococcus*	**this study**
Dictyochophyceae	*Florenciella*	<5	consumption and growth on *Prochlorococcu, Synechococcus*, bacteria	[8]; **this study**
	Rhizochromulina	~3	consumption and growth on *Prochlorococcus*	**this study**
	DictyX	3-5	consumption and growth on *Prochlorococcus*	**this study**
Raphidophyceae	*Heterosigma*	>10	ingestion of *Synechococcus*	[21]
**Cercozoa**				
Chlorarachniophyceae	*Cryptochlora*	>10	ingestion of eukaryotic prey	[22]
	Lotharella	>5	presence of food vacuole	[23]
	ChloraX	<5	consumption and growth on *Prochlorococcus*	**this study**
**Cryptophyta**				
Cryptophyceae	*Geminigera*	>5	ingestion of eukaryotic prey or microsphere	[24][13]
	Teleaulax	>5	ingestion of bacteria and *Synechococcus* sp.	[25]
**Haptophyta**				
Prymnesiophyceae	*Chrysochromulina*	>3	ingestion of bacteria or other prey	e.g. [26][27] **this study**
	Haptolina	>5	ingestion of bacteria or other prey	[28][2]
	Prymnesium	>5	ingestion of bacteria or other prey	e.g. [29][5]
incertae sedis	Hap2	~5	consumption and growth on *Prochlorococcus*	**this study**

†Taxa are mainly drawn from references [7][9][10] with original source references indicated. Specifically excluded are species that were isolated from benthic rocks or freshwaters, are larger than 20 µm, or for which evidence of bacterivory has been disputed (*Micromonas*, [11])

Supplementary References

1. Frias-Lopez J, Thompson A, Waldbauer J, Chisholm SW. Use of stable isotope-labelled cells to identify active grazers of picocyanobacteria in ocean surface waters. Environ Microbiol. 2009; 11: 512–525.
2. Hansen PJ, Hjorth M. Growth and grazing responses of *Chrysochromulina ericina* (Prymnesiophyceae): the role of irradiance, prey concentration and pH. Mar Biol. 2002; 141: 975–983.
3. Stibor H, Sommer U. Mixotrophy of a photosynthetic flagellate viewed from an optimal foraging perspective. Protist. 2003; 154: 91–98.
4. Burbage DC. Picocyanobacterial cellular physiology and trophic interaction with a heterotrophic nanoflagellate, Doctoral dissertation, University of Georgia at Athens. 2007.
5. Legrand C. Phagotrophy and toxicity variation in the mixotrophic *Prymnesium patelliferum* (Haptophyceae). Limnol Oceanogr. 2001; 46: 1208–1214.
6. Christaki U, Courties C, Karayanni H, Giannakourou A, Maravelias C, Kormas KA *et al*. Dynamic characteristics of *Prochlorococcus* and *Synechococcus* consumption by bacterivorous nanoflagellates. Microb Ecol. 2002; 43: 341–352.
7. Hansen PJ, Bjømsen PK, Hansen BW. Zooplankton grazing and growth: Scaling within the 2-2,000-µm body size range. Limnol Oceanogr. 1997; 42: 687–704.
8. Li Q, Edwards KF, Schvarcz CR, Selph KE, Steward GF. Plasticity in the grazing ecophysiology of *Florenciella* (Dictyochophyceae), a mixotrophic nanoflagellate that consumes *Prochlorococcus* and other bacteria. Limnol Oceanogr. 2021; 66: 47-60 e-pub; DOI: 10.1002/lno.11585.
9. Leles SG, Mitra A, Flynn KJ, Tillmann U, Stoecker D, Jeong HJ *et al*. Sampling bias misrepresents the biogeographical significance of constitutive mixotrophs across global oceans. Glob Ecol Biogeogr. 2019; 28: 418–428.
10. Anderson R, Charvet S, Hansen PJ. Mixotrophy in chlorophytes and haptophytes — effect of irradiance, macronutrient, micronutrient and vitamin limitation. Front Microbiol. 2018; e-pub; DOI:10.3389/fmicb.2018.01704.
11. Jimenez V, Burns JA, Le Gall F, Not F, Vaulot D. No evidence of Phago-mixotropy in *Micromonas polaris* (Mamiellophyceae), the Dominant Picophytoplankton Species in the Arctic. J Phycol. 2021; 57: 435–446.
12. Bock NA, Charvet S, Burns J, Gyaltshen Y, Rozenberg A, Duhamel S *et al*. Experimental identification and in silico prediction of bacterivory in green algae. ISMEJ. 2021; e-pub; https://doi.org/10.1038/s41396-021-00899-w.
13. McKie-Krisberg ZM, Gast RJ, Sanders RW. Physiological responses of three species of Antarctic mxotrophic phytoflagellates to changes in light and dissolved nutrients. Microb Ecol. 2014; 70: 21–29.
14. Anderson R, Jürgens K, Hansen PJ. Mixotrophic phytoflagellate bacterivory field measurements strongly biased by standard approaches: a case study. Front Microbiol. 2017; e-pub; DOI:10.3389/fmicb.2017.01398.

15. Maruyama S, Kim E. A modern descendant of early green algal phagotrophs. Curr Biol. 2013; 23: 1081–1084.

16. Bell EM, Laybourn-Parry J. Mixotrophy in the antarctic phytoflagellate, *Pyramimonas gelidicola* (Chlorophyta: Prasinophyceae). J Phycol. 2003; 39: 644–649.

17. Andersson A, Falk S, Samuelsson G, Hagström Å. Nutritional characteristics of a mixotrophic nanoflagellate, *Ochromonas* sp. Microb Ecol. 1989; 17: 251–262.

18. Sanders RW, Caron DA, Davidson JM, Dennett MR, Moran DM. Nutrient acquisition and population growth of a mixotrophic alga in axenic and bacterized cultures. Microb Ecol. 2001; 42: 513–523.

19. McKenrie CH, Deibel D, Paranjape MA, Thompson RJ. The marine mixotroph *Dinobryon balticum* (Chrysophyceae): phagotrophy and survival in a cold ocean. J Phycol. 1995; 31: 19–24.

20. Unrein F, Gasol JM, Massana R. *Dinobryon faculiferum* (Chrysophyta) in coastal Mediterranean seawater: Presence and grazing impact on bacteria. J Plankton Res. 2010; 32: 559–564.

21. Jeong JH, Seong KA, Kang NS, Yoo YD, Nam SW, Park JY *et al*. Feeding by raphidophytes on the cyanobacterium *Synechococcus* sp. Aquat Microb Ecol. 2010; 58: 181–195.

22. Calderon-Saenz E, Schnetter R. Morphology, biology and systematics of *Cryptochlora perforans* (Chlorarachniophyta), a phagotrophic marine alga. Plant Sys Evol. 1989; 163: 165–176.

23. Ota S, Silver TD, Archibald JM, Ishida K-I. *Lotharella oceanica* sp. nov. - a new plamktonic chlorarachniophyte studied by light and electron microscopy. Phycologia. 2009; 48: 315-323.

24. Gast RJ, McKie-Krisberg ZM, Fay SA, Rose JM, Sanders RW. Antarctic mixotrophic protist abundances by microscopy and molecular methods. FEMS Microbiol Ecol. 2014; 89: 388–401.

25. Yoo YD, Seong KA, Jeong HJ, Yih W, Rho JR, Nam SW *et al*. Mixotrophy in the marine red-tide cryptophyte *Teleaulax amphioxeia* and ingestion and grazing impact of cryptophytes on natural populations of bacteria in Korean coastal waters. Harmful Algae. 2017; 68: 105–117.

26. Jones HLJ, Leadbeater BSC, Green JC. Mixotrophy in marine species of *Chrysochromulina* (Prymnesiophyceae): ingestion and digestion of a small green flagellate. J Mar Biol Assoc United Kingdom 1993; 73: 283–296.

27. Edvardsen B, Paasche E. Bloom dynamics and physiology of *Prymnesium* and *Chrysochromulina*. NATO ASI Series G Ecol Sci. 1998; 41: 193–208.

28. Kawachi M, Inouye I, Maeda O, Chihara M. The haptonema as a food-capturing device: observations on *Chrysochromulina hirta* (Prymnesiophyceae). Phycologia. 1991; 30: 563–573.

29. Nygaard K, Tobiesen A. Bacterivory in algae: A survival strategy during nutrient limitation. Limnol Oceanogr. 1993; 38: 273–279.
